# A miniaturized-tumor culture platform for developing anti-tumor immunotherapies

**DOI:** 10.7150/thno.132113

**Published:** 2026-07-13

**Authors:** Yangyang Feng, Ling Li, Josh Haipeng Lei, Yuzhong Peng, Lijian Wang, Heng Sun, Dongyang Tang, Xinyu Guo, Xiangpeng Chu, Yunfeng Qiao, Kai Miao, Wen-Li Zhu, Chon-Wa Lam, Kin-Iong Chan, Wai-Kuok Chu, Shi-Xian Yao, Wei-Jian Hou, Xiaoling Xu, Zheng Chen, Chu-Xia Deng

**Affiliations:** 1 Cancer Center, Faculty of Health Sciences, University of Macau, Macau SAR 999078, China.; 2 Center for Precision Medicine Research and Training, Faculty of Health Sciences, University of Macau, Macau SAR 999078, China.; 3 MoE Frontiers Science Center for Precision Oncology, University of Macau, Macau SAR 999078, China.; 4 Kiang Wu Hospital, Macau SAR 999078, China.; 5 Zhuhai UM Science & Technology Research Institute, Hengqin, Zhuhai, Guangdong 519031, China.; 6 Department of Gastrointestinal Surgery, The Fifth Affiliated Hospital of Sun Yat-sen University, Zhuhai, Guangdong 519000, China.; 7 Faculty of Synthetic Biology, Shenzhen University of Advanced Technology, Shenzhen 518107, China.

**Keywords:** miniaturized-tumor culture, tumor immune microenvironment, immune checkpoint blockade, high-throughput drug screening, combination immunotherapy

## Abstract

**Rationale:**

The preservation of the tumor immune microenvironment (TIME) *ex vivo* is essential for investigating tumor-immune interactions and developing effective immunotherapies. However, current culture models often fail to maintain autologous immune cells or support high-throughput testing. To overcome these limitations, we establish and validate a novel miniaturized-tumor culture (MTC) platform.

**Methods:**

To build the MTC platform, we took tumor tissues from both mouse models and human patients. We processed these tissues into 100–500 μm fragments. Supplementation with IL-2 and IL-7 in culture maintained long-term intra-tumoral T cell survival. We then evaluated whether the platform could maintain autologous lymphoid populations and respond to immune checkpoint blockade (ICB), through co-culture assays with peripheral blood mononuclear cells (PBMCs) or splenocytes. The system accurately recapitulates drug response and resistance in both immunocompetent and immunodeficient models. Finally, we screened a high-throughput drug library to identify agents that re-sensitize tumors to anti-PD-L1 therapy. To figure out the underlying mechanisms, we used bulk RNA-sequencing, flow cytometry, and targeted CXCL13 antibody neutralization.

**Results:**

The MTC platform demonstrated better preservation of autologous lymphoid populations and remained responsive to ICB. Co-culture assays revealed enhanced immune cell infiltration upon ICB treatment. Furthermore, the MTC models recapitulated drug response and resistance phenotypes. Our screen identified axitinib (AXI) as the most potent agent for re-sensitizing tumors to anti-PD-L1 therapy across different cancer types. Mechanistically, AXI potentiates antigen presentation in tumor and dendritic cells. It also enhances cytotoxic T-cell function via the upregulation of CXCL13. Finally, CXCL13 blockade effectively abrogated AXI-induced T cell recruitment and tumor regression.

**Conclusions:**

The MTC platform serves as a high-fidelity, high-throughput tool for modeling the TIME. It provides a valuable framework for developing novel anti-cancer strategies and elucidating their underlying mechanisms of action.

## Introduction

Immune checkpoint blockade (ICB) has revolutionized cancer therapy, providing durable clinical responses in a subset of patients across various malignancies. By targeting inhibitory receptors such as PD-L1, PD-1 and CTLA-4, ICB antibodies reinvigorate pre-existing anti-tumor T cell responses, leading to tumor regression 1, 2. However, the majority of patients do not benefit from the ICB, primarily due to innate or acquired resistance. The mechanisms of resistance are multifaceted and can be broadly categorized into tumor-intrinsic and microenvironmental factors. Tumor-intrinsic mechanisms include defects in antigen presentation (*e.g.*, loss of MHC class I expression), alterations in interferon-gamma signaling pathways, and activation of alternative oncogenic cascades (*e.g.*, PI3K, WNT/β-catenin, MAPK) that suppress T cell recruitment 3-7. Within the tumor microenvironment (TME), the exclusion of cytotoxic T cells, the accumulation of immunosuppressive cells (such as Tregs, myeloid-derived suppressor cells, and M2-like macrophages), and the compensatory upregulation of alternative immune checkpoints (*e.g.*, LAG-3, TIM-3) further weaken an effective anti-tumor response 7-11. This complexity forces us to develop effective combination strategies. Such strategies are essential to overcome resistance and improve the therapeutic efficacy of ICB.

The world is moving away from animal testing in preclinical research. This is because of ethical concerns and the fact that animal models often fail to predict human responses. Notably, over 90% of drugs proven safe in animals still fail in human clinical trials 12. Regulatory agencies worldwide are now promoting New Approach Methodologies (NAMs), which include advanced in vitro systems, organ-on-a-chip platforms, computational modeling, and *ex vivo* human tissue models 13. In 2025, the U.S. FDA put out a plan to end conventional animal testing, starting with monoclonal antibodies. They also supported integrated safety assessments combining NAMs with real-world human data 14. Likewise, the UK government set deadlines to reduce or eliminate animal testing for drugs and chemicals 15. These indicate the important role of *ex vivo* models—not only in reducing animal use but also in accelerating personalized cancer therapies and improving translational outcomes. Since pharmaceutical pipelines are increasingly using these platforms, *ex vivo* models are becoming indispensable for ethical, efficient, and clinically relevant drug development.

To accelerate the discovery of novel immunotherapies, we need to develop *ex vivo* models that accurately recapitulate the human tumor immune microenvironment. Traditional cell lines and even patient-derived organoids often fail to retain the autologous immune compartment, reducing their value for immuno-oncology studies 16, 17. To solve this problem, a variety of organotypic and TME-preserving *ex vivo* platforms, such as tumor fragment cultures, microfluidic culture, air-liquid interface culture, organoid-PBMC co-culture or slice cultures have been developed to better maintain native cellular heterogeneity and immune content 18-22. However, these platforms still have significant limitations. Many of these systems experience rapid immune cell death, particularly of T lymphocytes. Under standard culture conditions, the immune cells lose function within days. Furthermore, the large size, heterogeneity, and technical complexity of many TME-preserving platforms limit their reproducibility and scalability. They are not suitable for systematic, high-throughput drug screening and comprehensive evaluation of immune-dependent therapeutic responses.

Beyond the challenges of maintaining immune cell viability, existing *ex vivo* platforms are often poor at revealing the underlying mechanisms of immune regulation. Standard cultures often fail to preserve the spatial architecture and dynamic interactions, which are essential for immune function. Consequently, these models often fail to fully capture the complex immune responses to therapy—including T cell priming, recruitment, infiltration, and cytotoxic killing. This impedes not only drug screening but also the elucidation of mechanistic insights that are essential for designing the next generation of immunotherapies. Therefore, we urgently need an *ex vivo* platform that can both maintain a functional TME for drug evaluation and serve as a discovery tool for exploring the mechanisms of immune response and resistance.

To overcome these critical limitations, we developed the miniaturized-tumor culture (MTC) platform. It preserves both the viability and functionality of autologous immune populations, including T cells, derived from multiple cancer types. Its scalability is sufficient for high-throughput drug screening, allowing for the systematic identification of novel combination therapies. Using the MTC platform, we successfully screened ICB synergistic drugs from a 90-drug library and validated them in more than 30 patient-derived specimens across various cancer types. Our findings demonstrate that these screened drugs can significantly enhance ICB response. The MTC platform therefore represents a significant advance. It bridges the gap between high-throughput drug discovery and a deeper understanding of immune modulation within the native tumor microenvironment.

## Materials and Methods

### Human material

Human cancer tissues and peripheral blood samples were collected from patients with a confirmed pathological diagnosis of various cancer types. Surgical resection or biopsy specimens were obtained from these patients at Kiang Wu Hospital and The Fifth Affiliated Hospital of Sun Yat-sen University. The use of human material in this study was approved by the ethical review boards of the University of Macau and both hospitals under approval number BSER-E16-APP010-FHS.

### Mice

Female BALB/c, C57BL/6J, Nude, and FVB mice were used at the age of 6-8 weeks. *Neu*/*Fgfr2*-mutant (*Erbb2*^+^; *Fgfr2^pLoxpneo-S252W/+^, MMTV-Cre*), *p53*/*Fgfr2*-mutant (*p53*^-/-^; *Fgfr2^pLoxpneo-S252W/+^, MMTV-Cre*),* Pten-*mutant (*Pten*^-/-^; *MMTV-Cre*) and* Brca1*^co/+^; *MMTV-Cre* mice spontaneously developed tumor at the age of 12-24 months. All mice were housed under specific pathogen–free (SPF) conditions. The facility was strictly controlled for temperature and humidity, maintaining a 12 h light/dark cycle. Food and water were available ad libitum, and animals were housed on autoclaved corn cob bedding. All procedures complied with institutional guidelines and approved by the University of Macau Animal Ethics Committee (Accreditation number: UMARE-015-2019).

### Tumor models and *in vivo* treatments

For mouse tumor model establishment, mice were first anesthetized. Next, 1 × 10^6^ MC38, Hepa1-6, MC38-OVA, B16-F10 or LLC cells were suspended in 100 µL of PBS and was delivered via subcutaneous (s.c.) injection. 4T1, EMT6 or HP10069 cells were implanted into the mammary fat pads of BALB/c mice, Nude mice, or FVB mice (6–8 weeks old), and each mammary fat pad was injected with 5 × 10^5^ cells in 100 µL of PBS. MC38, Hepa1-6, LLC, HP10069 and MC38-OVA cell lines were cultured at 37 ºC in 5% CO_2_ in DMEM supplemented with 10% FBS and 1% penicillin-streptomycin. B16-F10 cells were cultured in DMEM/F12 supplemented with 10% FBS and 1% penicillin-streptomycin. 4T1 and EMT6 cells were cultured in RPMI1640 supplemented with 10% FBS and 1% penicillin-streptomycin. Mice bearing 4T1 or EMT6 tumors were randomly assigned to one of the following treatments: doxorubicin (2 mg/kg), epirubicin (2 mg/kg), gemcitabine (10 mg/kg), or paclitaxel (10 mg/kg) via intraperitoneal (i.p.) injection every 3 days; axitinib (25 mg/kg) daily by oral gavage; or regorafenib (5 mg/kg) every other day by oral gavage. Tumor volumes were calculated using the formula: (length × width^2^)/2, based on dimensions taken every 3 days with a digital caliper.

### Construction and optimization of MTC model

Mouse tumor specimens were collected into ice-cold DMEM with 10% FBS and 1% penicillin-streptomycin, and surgically resected human tumor samples were collected into ice-cold Advanced DMEM/F12 with 10% FBS and 1% penicillin-streptomycin. Tumors were typically obtained at millimeter-to-centimeter scale prior to processing. Tissues were mechanically minced on a 500-μm wire mesh in a 10-cm dish using sterile forceps and scissors to generate fragments smaller than 500 μm. The resulting tissue fraction was pelleted and resuspended in fresh medium, then filtered through 100-μm and 40-μm strainers. This produced two defined size ranges (S1: 40–100 μm; S2: 100–500 μm), ensuring reproducible size classification without manual cutting. To reduce the impact of intratumoral heterogeneity, tissues were extensively minced and mixed prior to size separation. The S2 fraction was collected, diluted in medium, and the number of fragments was quantified under a microscope. Multiple fragments (typically 40–60 per well) were used for each experimental condition.

An ice-cold collagen solution was generated by mixing three components in an 8:1:1 volume ratio: solution A (rat collagen I from R&D Systems, 3447-020-01), solution B (10× Ham's F-12), and solution C (a sterile reconstitution buffer containing 2.2 g NaHCO3 in 100 mL of 0.05 M NaOH and 200 mM HEPES). The mixture was then diluted with 1 × Ham's F-12 to a final concentration of 2.4 mg/mL. Next, 100 μL of the collagen solution was placed into the inserts (37524, SPLInsert Standing; or TCS016024, Biofil) and incubated at 37 °C for 20–30 min until the collagen was fully coagulated. The S2 fractions of tumor tissues were then mixed with the ice-cold collagen solution, and 50 μL of this tissue suspension, containing 40-60 fragments, was added to the respective inserts. After the top tissue-gel layer had set, the outer chambers of the culture inserts received 350 μL of basal or drug-supplemented medium. Plates were then placed in a humidified incubator at 37 °C and 5% CO_2_ for the indicated duration.

The basal medium used for mouse tumors was F-medium 23, prepared by mixing 250 mL of DMEM and 250 mL of DMEM/F-12 Nutrient Mix. This mixture was supplemented with 5 μg/mL insulin, 250 ng/mL amphotericin B, 10 μg/mL gentamicin, 0.1 nM cholera toxin (C8052, Sigma-Aldrich), 0.125 ng/mL EGF (AF-100-15, PeproTech), 25 ng/mL hydrocortisone (H3506, Sigma-Aldrich), and 10 μM ROCK inhibitor Y-27632 (DC1028, DC Chemicals). The composition of the medium for human tumor samples was adapted from previously established formulations, with slight modifications 24, 25. For human breast cancer MTC models, Advanced DMEM/F12 was supplemented with 30% L-WRN conditioned medium, 5 μM Y-27632, 0.5 μM SB202190 (DC2097, DC Chemicals), 0.5 μM A83-01 (DC7286, DC Chemicals), 5 ng/mL EGF, 500 ng/mL hydrocortisone, 10 mM nicotinamide (N3376, Sigma-Aldrich), 1.25 mM N-acetyl-L-cysteine (A9165, Sigma-Aldrich), 15 mM HEPES, 1 × B27 (17504-001, GIBCO), 1 × GlutaMAX (35050-061, GIBCO), 1 × insulin-transferrin-selenium-sodium pyruvate (51300044, GIBCO), 0.5 μg/mL amphotericin B (15290-018, GIBCO), 5 μg/mL gentamicin, 5 μg/mL plasmocin, 5 nM β-Estradiol (E2758, Sigma-Aldrich) and 5 nM Neuregulin-1 (100-03, PeproTech). The culture media used for the human colon cancer, lung cancer and nasopharyngeal cancer were Advanced DMEM/F12 supplemented with 30% L-WRN conditioned medium, 10 μM Y-27632, 10 μM SB202190, 0.5 μM A83-01, 50 ng/mL EGF, 500 ng/mL hydrocortisone, 10 mM nicotinamide, 1.25 mM N-acetyl-L-cysteine, 15 mM HEPES, 1 × B27, 1 × GlutaMAX, 1 × insulin-transferrin-selenium-sodium pyruvate, 0.5 μg/mL amphotericin B, 5 μg/mL gentamicin, and 5 μg/mL plasmocin. The culture medium for human liver cancer was Advanced DMEM/F12 supplemented with 30% L-WRN conditioned medium, 10 μM Y-27632, 10 μM SB202190, 0.5 μM A83-01, 50 ng/mL EGF, 10 mM nicotinamide, 1.25 mM N-acetyl-L-cysteine, 20 mM HEPES, 1 × B27, 1 × N2 (17502-048, GIBCO), 10 nM [Leu15]-Gastrin I human (G9145, Sigma-Aldrich), 10 ng/mL HGF (100-39, PeproTech), 5 μM Forskolin (6652995, PeproTech), 1 × GlutaMAX, 0.5 μg/mL amphotericin B, 5 μg/mL gentamicin and 5 μg/mL plasmocin. To sustain immune cell viability, all functional media were further supplemented with 500 U/mL recombinant IL-2 (212-12 for mouse, 200-02 for human; PeproTech) and either 10 or 100 ng/mL recombinant IL-7 (217-17 for mouse, 200-07 for human; PeproTech).

### Quantitative evaluation of drug sensitivity

After the indicated drug treatments ended, MTT solution (400 μL per insert; final concentration 0.25 mg/mL) was added to each MTC insert, and the inserts were left to incubate at 37 °C for 4 h. Tissue-containing top layers were subsequently relocated to a 6-well plate, where images were captured on a Leica M165 FC fluorescent stereo microscope. The tissues were then transferred to a 96-well plate, where 100 μL of DMSO was used to dissolve the formazan crystals. The absorbance of the resulting solution was then measured at a wavelength of 570 nm.

For high-throughput drug screening to determine the IC_50_ of MTC models with or without intact TIME, mouse MTCs diluted in collagen solution were dispersed into a 48-well plate with 50 μL/well. After the gel solidified, F-medium containing the indicated drugs with different concentrations (starting at 100 µM and serially diluted 4-fold for 5 steps) was added into the plate, and the MTCs were cultured for 4 days. For high-throughput drug screening of drugs that synergize with ICB, once the gel had solidified, F-medium containing IgG or 5 µg/mL anti-PD-L1 (124302, BioLegend), with or without the indicated drugs (Table S1), was added into the plate and the MTCs were cultured for 4 days. At the endpoint of culturing, the medium containing drugs were aspirated, and each well was added with 200 μL of MTT solution for incubation of 4 h. After that, the tissues were transferred to a 96-well plate, where 100 μL of DMSO was added to dissolve the formazan in each well. The absorbance of each well was measured at a wavelength of 570 nm.

For validation of drug synergy with anti-PD-L1, MTC models were treated with anti-PD-L1 (10 µg/mL; 124302, BioLegend for mouse; Atezolizumab, HY-P9904, MCE for human) either alone or in combination with axitinib (AXI, 1 or 2 µM, HY-10065, MCE), ruxolitinib (RUX, 20 µM, HY-50856, MCE), chlorambucil (CHL, 20 µM, HY-13593, MCE), or rosiglitazone (ROS, 20 µM, HY-17386, MCE) for 4 days, followed by assessment of cell viability using the MTT assay and immune cell activity by flow cytometry. Where indicated, cultures were treated with a CXCL13-neutralizing antibody (1 µg/mL; MAB470, R&D Systems), anti-PD-1 (10 µg/mL; 135248, BioLegend) or anti-CTLA-4 (10 µg/mL; 106214, BioLegend).

For viability fluorescence assessment, the MTC models were first washed with PBS. They were then stained using a mixture of AO/PI Staining Solutions (CS2-0106, Nexcelom) or Calcein-AM/PI (Invitrogen) and Hoechst 33342 (10 μg/mL, Invitrogen). The models were incubated with the dyes in the dark for 30 min at 37 ºC. Fluorescence images were then acquired using a Nikon A1R laser scanning confocal microscope equipped with a 10x objective. The NIS-Elements AR software package was used for all image acquisition and subsequent data analysis.

### Isolation of PBMCs and splenic lymphocytes

Human blood samples were obtained via venipuncture, whereas mouse blood was collected through retro-orbital sinus bleeding. PBMCs were then isolated by Ficoll-Paque™ PREMIUM (1.084) density gradient centrifugation. Lymphocytes from the spleen were isolated by gently pressing the spleen through a 40 μm strainer.

### Dendritic cell–T cell co-culture assay

DC2.4 cells were first pretreated with axitinib (AXI, 1 or 2 µM) for 24 h to modulate antigen-presenting function. Following pretreatment, cells were washed to remove residual drug and then co-cultured with purified CD8⁺ T cells at a 1:10 ratio. CD8⁺ T cells were isolated using a mouse CD8⁺ T cell isolation kit (19853A, STEMCELL Technologies) and pre-activated with Dynabeads Mouse T-Activator CD3/CD28 (11452D, Gibco) for 3 days prior to co-culture; beads were removed before co-culture with DC2.4 cells. The co-culture was maintained for 48 h to allow dendritic cell–mediated T-cell priming and activation. After incubation, T cells were harvested and analyzed by flow cytometry to assess activation markers, including CD69 expression on CD8⁺ T cells.

### Flow cytometry analysis

Tumor tissues from MTC models were collected and single cells were prepared by dissociation with 0.5 mg/mL collagenase II (LS004176, Worthington Biochemicals) and 1 mg/mL collagenase IV (LS004188, Worthington Biochemicals) at 37 ºC for 30 min, followed by three 5-min washes with DMEM medium. For co-culturing of MTC and PBMCs or splenocytes, cells that failed to enter the MTC were removed by washing twice with PBS.

To prevent non-specific binding, mouse Fc receptors were blocked using a purified anti-mouse CD16/CD32 blocking antibody (70-0161, Tonbo Biosciences) for 15 min at 4 ºC. Cells were then incubated with the following surface antibodies: CD45-APC (103112, BioLegend), CD45-Brilliant Violet 421 (103133, BioLegend), CD3-FITC (100204, BioLegend), CD4-PE/Cy7 (100422, BioLegend), CD8-PerCP/eFluor 710 (46-0081-82, Invitrogen), CD44-Alexa Fluor 700 (103026, BioLegend), CD25-Brilliant Violet 605 (102036, BioLegend), CD69-Brilliant Violet 421 (104527, BioLegend), ICOS-Brilliant Violet 650 (313550, BioLegend) and a Live/Dead Fixable Near-IR viability dye (L10119, Invitrogen) in FACS buffer (PBS supplemented with 2% FBS and 2 mM EDTA) for 30 min at 4 ºC. For intracellular staining of secreted proteins, a Protein Transport Inhibitor Cocktail (00-4980-03, Invitrogen) was added 4 h before sample collection. After cell-surface staining, cells were fixed in 4% PFA for 10 min and stained for intracellular IFNγ-PE (505808, BioLegend) or CXCL13-APC (17-7981-82, Invitrogen) using 0.05% Triton X-100 for 30 min. For human samples, Fc receptors were blocked before the addition of cell surface antibodies. This step was performed using a Human FcR Blocking Reagent (564220, BD) for 15 min at 4 ºC. Cells were then incubated with Zombie Violet viability dye (423117, BioLegend) in PBS for 30 min at 4 ºC, followed by staining with CD45-PE/Cy7, CD3-BV510, and CD8-R718 (from the BD Horizon™ Human T Cell Backbone Panel, 568263, BD) and CD4-FITC (FITC-98042, Proteintech) in FACS buffer for 30 min at 4 ºC. Cells were washed and resuspended in FACS buffer and analyzed using a Cytoflex S flow cytometer. Data were analyzed using FlowJo software version 10.8.1.

### Whole-mount immunofluorescence for MTC

For immunofluorescence studies, MTCs were washed with PBS and fixed with 4% PFA for 15 min at room temperature. The PFA was then washed away with PBS and a PBS-Glycine solution (PBS containing 0.1 M glycine) for 10 min at room temperature. For permeabilization, the PBS-Glycine solution was aspirated and a PBT solution (PBS containing 0.1% Tween 20) was added for 10 min at room temperature. For blocking, the PBT was removed and a PBST solution (PBS containing 0.1% Triton X-100 and 0.2% BSA) was added for 15 min at room temperature. Directly conjugated antibodies: CD45-AlexaFluor488 (103122, BioLegend), CD8a-PE (100708, BioLegend), CD11b-FITC (101206, BioLegend), and F4/80-APC (17-4801-80, eBioscience) were then added at a 1:400 dilution in PBST solution and incubated overnight at 4 ºC. For unconjugated staining, α-SMA (ab124964, Abcam) was added at 1:400 dilution in PBST and incubated overnight at 4 ºC. Following two washes with PBST, the samples were incubated with goat anti-rabbit IgG (H+L) Alexa Fluor 594 (1:500, A-11012, Thermo Fisher Scientific) overnight at 4 ºC. The samples were then washed twice with PBST solution, followed by incubation with a clearing solution (60% (vol/vol) glycerol and 2.5 M fructose) containing 10 μg/mL Hoechst 33342 (Thermo Fisher Scientific) for 20 min at room temperature in the dark. Images were then captured on a Nikon A1R laser scanning confocal microscope. A 60x silicone-oil objective lens was used for the imaging. Images were taken and analyzed with the NIS-Elements AR software package.

To perform immune cell infiltration tracking, splenocytes and MTCs were pre-labeled with distinct fluorescent dyes. Splenocytes were incubated with 5 μM CFSE (C1157, Invitrogen) and MTCs with 5 μM CellTrace Violet (C34557, Invitrogen) in PBS for 20 min at 37 ºC. The staining was subsequently quenched with FBS, and the cells were washed with PBS. Images of the MTC co-cultures were taken on a Nikon A1R confocal microscope using a 20x objective.

### RNA sequencing and data analysis

TRIzol reagent was used to extract RNA from MTCs. Sample quantity, integrity, and purity were assessed using agarose gel electrophoresis. The total RNA amount in each sample exceeded 40 µg. Samples were sent to Beijing Youji Technology for library preparation and sequencing. Adapter-contaminated reads (N>10%) and low-quality reads were removed before further processing; quality was then checked with MultiQC 26. Clean reads were mapped onto the GRCm39 mouse reference genome using STAR 27, and uniquely mapped reads were summarized using FeatureCounts 28. Differentially expressed genes (DEGs) were identified with DESeq2, with filtering criteria set at a minimum 2-fold change and an FDR-adjusted p-value of 0.05.

### Statistical analysis

Data plotting and statistical analyses were performed using GraphPad Prism 10. Differences between two groups were analyzed using an unpaired, two-tailed Student’s t tests. Differences among more than two groups were analyzed by two-way ANOVA followed by appropriate post hoc multiple comparison tests, including Dunnett’s test (for comparisons versus a control group), Tukey’s test (for all pairwise group comparisons), or Sidak’s test (for selected multiple comparisons), as appropriate. Statistical significance was defined as follows: *p < 0.05, **p < 0.01, ***p < 0.001, and ****p < 0.0001; “ns” indicates no significant difference. Data in column bar plots are displayed as mean ± standard deviation (SD) in all bar plots unless specified otherwise. FlowJo software was used for flow cytometry data analysis. Microscopy images were processed and quantified via ImageJ. Transcriptome data processing is described in detail in the preceding sections.

## Results

### Generation of a miniaturized-tumor culture model preserving the integrity of the tumor immune microenvironment

**Figure 1:**
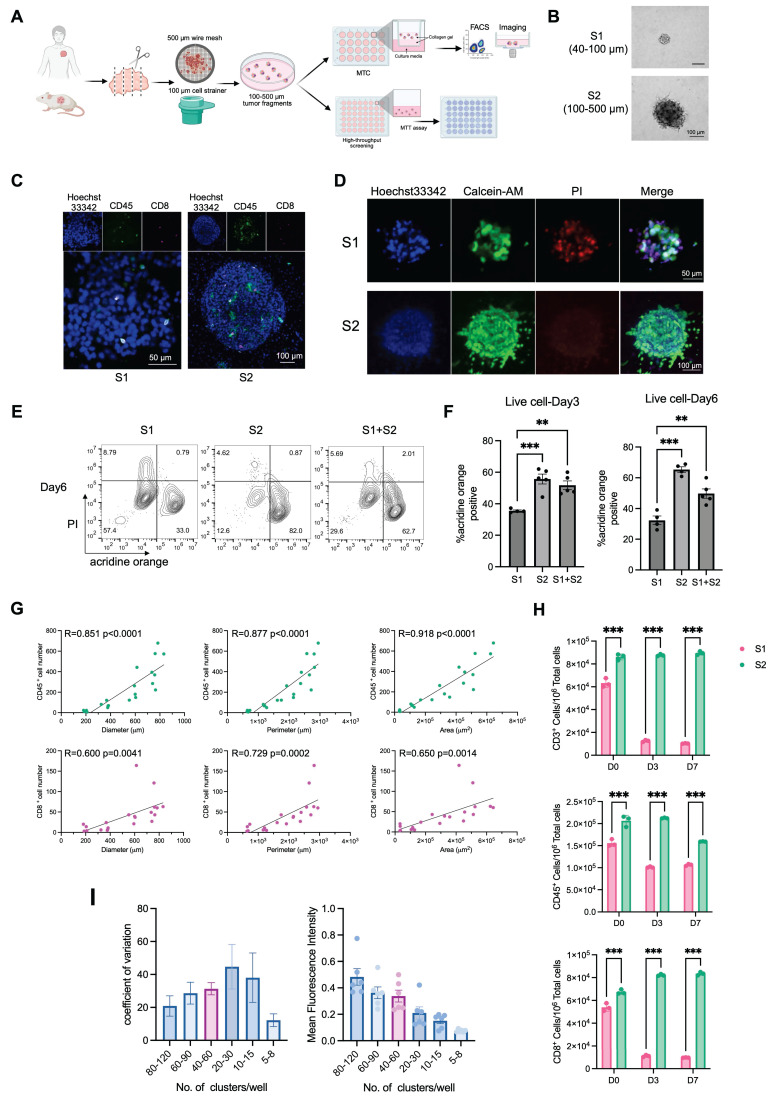
** Establishment and optimization of the MTC model.** (A) Schematic diagram illustrating the procedure of MTC model. Created in BioRender. Von, Y. (2026) https://BioRender.com/or51j3k. (B) Representative phase-contrast images of EMT6 tumor fragments of different sizes cultured in the MTC system. Scale bars are shown. (C) Representative immunofluorescence images of MTC-cultured tissues stained for CD45⁺ immune cells (green) and CD8⁺ T cells (magenta) after 3 days in culture. (D) Live/dead staining of EMT6 MTC models with different fragment sizes on day 3. Viable cells are labeled with Calcein-AM (green); dead cells are labeled with propidium iodide (PI, red). Scale bars are indicated. (E) Flow cytometry analysis of live/dead cells in EMT6 MTC models with different fragment sizes on day 6. Acridine Orange (AO) for live cells; PI for dead cells. (F) Quantitative analysis of the proportion of live/dead cells by FACS on day 3 and day 6 (mean ± SD; n ≥ 4). (G) Correlation analysis between the numbers of CD45^+^ immune cells or CD8^+^ T cells and the diameter, perimeter, or area of the MTC tumor fragments after 3 days of culture. R, correlation coefficient. (H) FACS quantitation of CD45^+^ immune cell, CD3^+^ T cell and CD8^+^ T cell number among 10^6^ live cells in differently sized MTCs from Hepa1-6 tumors at day 3 and day 7 (n = 3). (I) Analysis of MTT fluorescence intensity and its variation (coefficient of variation) across wells seeded with different numbers of tumor fragments (n = 6). Data are presented as mean ± SD; **p < 0.01; ***p < 0.001.

To establish a miniaturized-tumor culture model amenable to high-throughput drug screening, we developed a new system by extensively modifying the culture protocols (Figure 1A). Given that larger tissue fragments have the potential to better preserve the native tumor immune microenvironment, we compared immune cell composition and overall cell viability across fragments of varying sizes. Tumor tissues from mouse samples were minced into fragments with diameters under 500 μm or under 100 μm and subsequently separated into three distinct size groups: S1 (40–100 μm), S2 (100–500 μm), and S1+S2 (40–500 μm), which was initially included to assess whether mixing fragment sizes could balance immune cell content and tissue integrity (Figure 1B). Whole-mount immunofluorescence staining for CD45 and CD8 revealed that S2 fragments retained more immune cell populations than S1 fragments (Figure 1C). Furthermore, staining with the viability dyes Calcein-AM and propidium iodide (PI) indicated that S2 group had better cell viability than S1 group (Figure 1D). We also used flow cytometry to measure cell viability in cultured fragments at day 3 and day 6. We found that S2 fragments, as well as a combination of S1 and S2 fragments, maintained higher viability, highlighting the benefit of using larger fragment sizes (Figure 1E, F). Comparing both S1 and the combined S1+S2 condition, S2 fragments alone provided more stable preservation of viable tumor tissue and functional T cell populations (Figure S1A). Consistent with this, quantitative analysis demonstrated that the number of CD45⁺ immune cells and CD8⁺ T cells correlated positively with fragment size (Figure 1G). Flow cytometry analysis further showed that S2 fragments contained significantly higher proportions of total immune cells, CD3⁺ T cells, CD8⁺ T cells, and CD4⁺ T cells compared to S1 fragments (Figure 1H and Figure S1B). Thus, we selected S2 fragments for downstream experiments. In addition to T cells, other immune and stromal components were detected in the MTC system, including dendritic cells, B cells, NK cells, myofibroblastic cancer-associated fibroblasts (α-SMA⁺), and tumor-associated macrophages (CD11b⁺/F4/80⁺), indicating preservation of a heterogeneous tumor microenvironment. These populations were maintained during early culture, although macrophages and dendritic cells gradually declined over time (Figure S1C, D). Using this optimized system, we successfully established MTC models from human lung, colon, breast, nasopharyngeal, and liver cancer specimens (Figure S1E). We also compared between the MTC platform and conventional tumor organoid cultures. The results showed that MTCs better preserve endogenous T cells and support immune activation. Unlike organoids, which lose T cells rapidly, MTC models maintain CD8⁺ T cells over time and remain responsive to immune checkpoint blockade (Figure S1F, G). To enable the model for drug screening, we used 24-well plates with embedded inserts for culture and evaluated drug responses using the MTT assay. We also figured out the optimal fragment density per well. Seeding 40–60 fragments provided reliable detection signals and minimal well-to-well variability (Figure 1I). Taken together, these optimizations prove that the MTC platform is a reliable and scalable *ex vivo* system. It maintains a functional tumor-immune microenvironment and is suited for high-throughput drug discovery.

### IL-7 facilitates the long-term maintenance of T cell populations in MTC model

**Figure 2:**
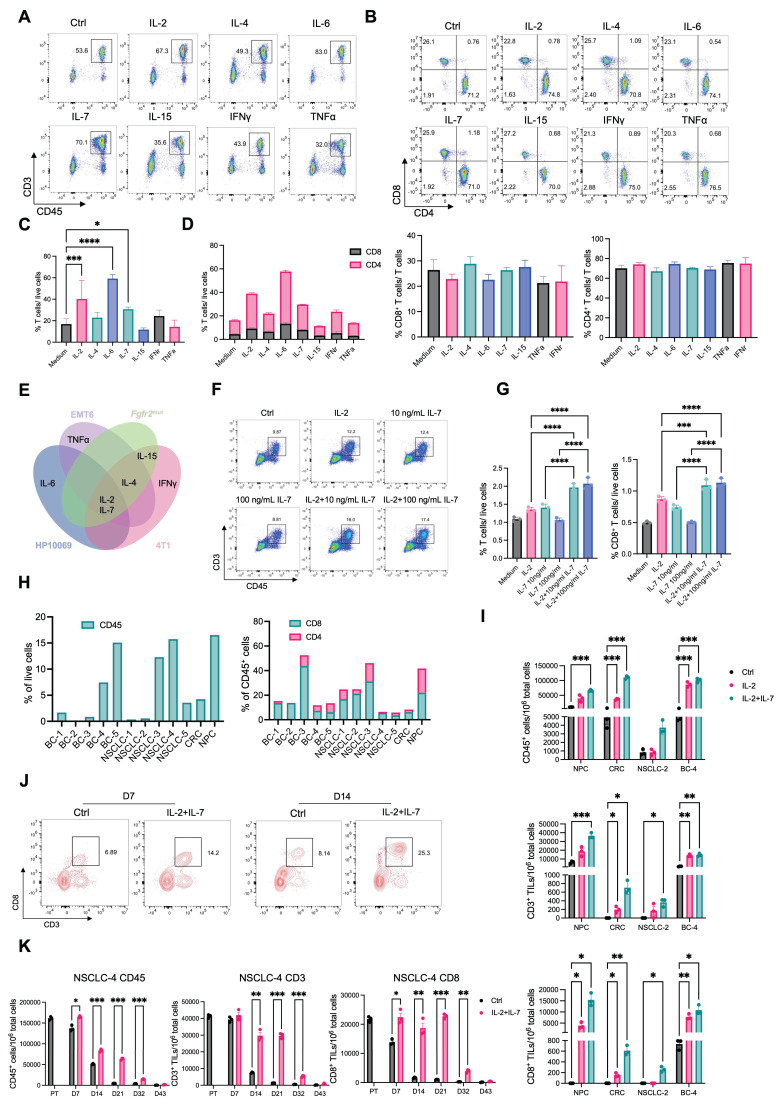
** IL-7 facilitates the long-term maintenance of T cell populations in MTC model.** (A) Representative flow cytometry analysis showing the proportion of T cells (gated on SSC-low, viable cells) in HP10069 MTC models treated with the indicated cytokines for 3 days. (B) Flow cytometry analysis of CD8^+^ T cell and CD4^+^ T cell subsets among CD3^+^ T cell population in HP10069 MTC models treated with the indicated cytokines for 3 days. (C) Quantification of T cell proportion among live cells in HP10069 MTC models treated with different cytokines for 3 days (n = 3). (D) Statistical analysis of CD8^+^ T cell and CD4^+^ T cell proportion among CD3^+^ T cells or live cells in HP10069 MTC models treated with different cytokines for 3 days (n = 3). (E) Venn diagram summarizing cytokines that enhanced T cell proportions across different MTC models at day 3. (F) Representative flow cytometry analysis showing the proportion of T cells (gated on SSC-low, viable cells) in MC38 MTC models treated with IL-2 combined with 10 ng/mL or 100 ng/mL IL-7 for 3 days. (G) Statistical analysis of CD8^+^ T cell and CD3^+^ T cell proportion among live cells in MC38 MTC models treated with IL-2 combined with 10 ng/mL or 100 ng/mL IL-7 for 3 days (n = 3). (H) Flow cytometry analysis of immune cell proportion among live cells or CD8^+^ T cell and CD4^+^ T cell proportion among immune cells in primary human tumor samples. (I) FACS quantitation of CD45^+^ immune cell, CD3^+^ T cell and CD8^+^ T cell number among 10^6^ live cells in human MTC models derived from different tumor types, cultured with or without IL-2 or IL-7 for 7 days (n = 3). (J) Representative flow cytometry plots showing CD8^+^ T cell frequency among immune cells in a human NSCLC sample (NSCLC-4). (K) FACS quantitation of CD45^+^ immune cell, CD3^+^ T cell and CD8^+^ T cell number among 10^6^ live cells in the NSCLC-4 human sample. NSCLC-4 MTC models were cultured with or without IL-2 and IL-7 (n = 3). Data are presented as mean ± SD; *p < 0.05; **p < 0.01; ***p < 0.001; ****p < 0.0001.

Previous studies have demonstrated that while air-liquid interface (ALI) cultures can initially maintain T cell populations, the number of these cells decreased substantially after several days in culture 22. To extend the persistence of T cells in the MTC system, we supplemented the culture medium with a panel of cytokines. IL-2, IL-4, IL-6, IL-7, IL-15, IFNγ, and TNFα were previously reported to support T cell proliferation and activation. We measured the frequencies of CD3⁺, CD8⁺, and CD4⁺ T cells among live cells in multiple tumor models, including 4T1, EMT6, and HP10069 implanted tumors, as well as *Fgfr2*-mutant endogenous tumors. The results indicated that cytokines supported T cell maintenance to varying degrees across different tumor models. Some cytokines, such as IL-4 and IL-15, exhibited model-specific or limited activity, whereas IL-2 and IL-7 consistently sustained T cell populations across all models without significantly altering the CD8⁺/CD4⁺ T cell ratio (Figure 2A-E and Figure S2A-H). We further validated the effects of IL-2 and IL-7 in the MC38 implanted colon cancer model, where we observed that the combination of IL-2 and IL-7 more effectively maintained T cell numbers compared to either cytokine alone, and that a concentration of 10 ng/mL IL-7 was sufficient to support T cell persistence (Figure 2F, G). In the absence of cytokine supplementation, T cell numbers in MTC models declined rapidly within 6 days; however, the addition of IL-2 and IL-7 enabled T cell preservation for at least 10 days (Figure S2I, J).

To examine whether IL-7 could similarly preserve T cells in human-derived samples, we established MTC models from 12 human cancer specimens, including breast, lung, colon, and nasopharyngeal carcinomas. Analysis of primary tumor tissues revealed considerable differences in immune cell composition across different samples (Figure 2H). Across diverse tumor types, IL-2 increased the proportions of total immune cells, CD3⁺ T cells, and CD8⁺ T cells. The combination of IL-2 and IL-7 produced the strongest effect (Figure 2I). We also evaluated the duration of immune and T cell preservation supported by these cytokines. Both populations could remain viable for more than two weeks, and CD8^+^ T cells could persist for up to 32 days in some cases (Figure 2J, K and Figure S2K).

To further examine whether IL-7 affects drug effects, we compared responses to ICB and axitinib (both require T-cell activity) under conditions with standard IL-7, reduced IL-7, or no IL-7 with only IL-2 supplementation. Significant differences in drug responses were not observed across these conditions (Figure S2L, M). This indicates that IL-7 primarily supports T-cell viability without altering treatment sensitivity in the MTC system. In addition, IL-7 is known to modulate JAK/STAT pathway. To evaluate its potential effects, we assessed ruxolitinib responses under different IL-7 conditions. We observed comparable antitumor effects regardless of IL-7 levels (Figure S2N). This suggests that IL-7–mediated signaling does not significantly influence JAK/STAT-targeted drug responses.

Together, these findings show that IL-7, alone or in combination with IL-2, is critical for sustaining functional T cell populations in the MTC platform. This extends the platform’s utility for long-term immuno-oncology studies.

### MTC model recapitulates *in vivo*-like drug response profiles

**Figure 3:**
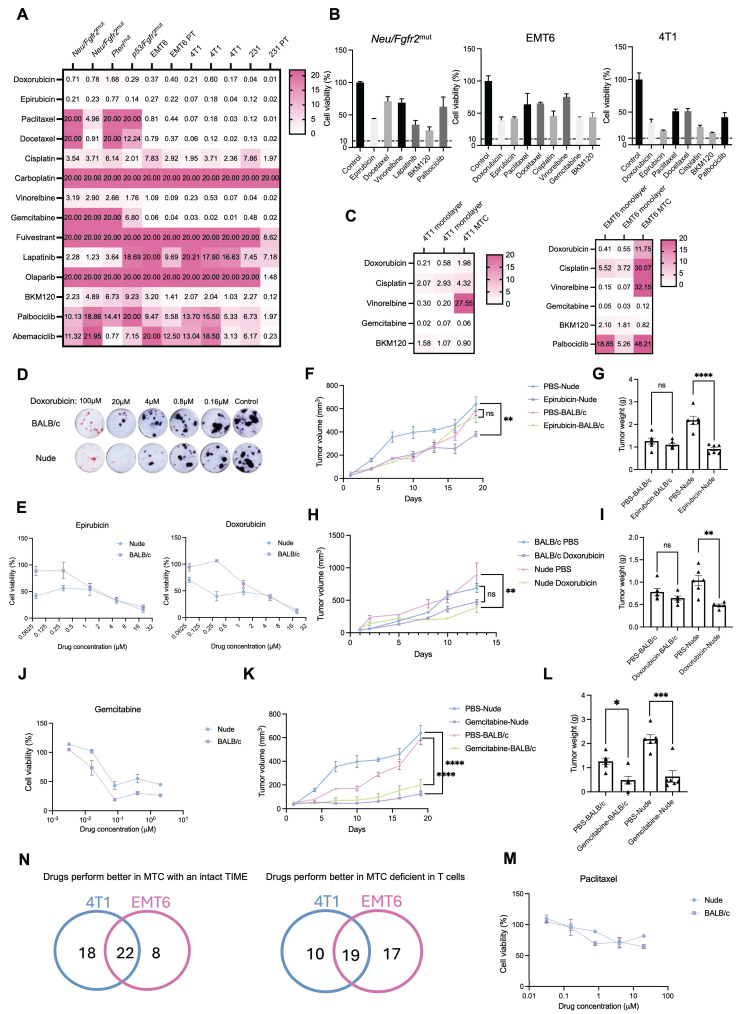
** MTC model recapitulates *in vivo*-like drug response profiles.** (A) Heatmap of IC_50_ values for the indicated drugs in monolayer (2D) cultures of different cells. (B) Cell viability of different MTC models treated with drugs at the IC_90_ concentration determined from 2D cultures (mean ± SD; n = 3). (C) Comparison of drug IC_50_ values between 2D cultures and MTC models. (D) Representative images of MTT staining in 4T1 breast tumor MTC models derived from immunodeficient (Nude) or immunocompetent (BALB/c) mice after treatment with the indicated concentrations of doxorubicin. (E) Dose-response curves of cell viability in 4T1 MTC models derived from Nude mice or BALB/c mice treated with epirubicin or doxorubicin (n ≥ 3). (F) Tumor growth curves of 4T1 cells implanted in Nude mice or BALB/c mice and treated with epirubicin (n = 6). (G) Comparison of final tumor weights in 4T1-bearing Nude or BALB/c mice treated with epirubicin (n = 6). (H) Tumor growth curves of 4T1 cells implanted in Nude mice or BALB/c mice and treated with doxorubicin (n ≥ 5). (I) Comparison of final tumor weights in 4T1-bearing Nude or BALB/c mice treated with doxorubicin (n ≥ 5). (J) Dose-response curves of cell viability in 4T1 MTC models derived from Nude mice or BALB/c mice treated with gemcitabine (n = 3). (K) Tumor growth curves of 4T1 cells implanted in Nude mice or BALB/c mice and treated with gemcitabine (n = 6). (L) Comparison of final tumor weights in 4T1-bearing Nude or BALB/c mice treated with gemcitabine (n = 6). (M) Dose-response curves of cell viability in 4T1 MTC models derived from Nude mice or BALB/c mice treated with paclitaxel (n = 3). (N) (Left) Venn diagram summarizing overlap of drugs that showed superior efficacy in MTC models derived from tumors grown in immunocompetent mice, between 4T1 and EMT6 models. (Right) Venn diagram summarizing overlap of drugs that showed superior efficacy in MTC models derived from tumors grown in immunodeficient mice, between 4T1 and EMT6 models. Data are presented as mean ± SEM; ns, not significant; *p<0.05; **p<0.01; ***p<0.001; ****p<0.0001.

A primary objective in developing the MTC model was to achieve more accurate prediction of drug responses. This platform preserves a more physiologically relevant tumor immune microenvironment compared to conventional 2D cell cultures, 3D organoids, or traditional ALI cultures. We therefore compared drug responses between 2D cultures and the MTC system, analyzing their respective half-maximal inhibitory concentration (IC_50_) values. We derived cancer cells from implanted and endogenous mouse tumors and tested a panel of clinically relevant drugs (Figure 3A). MTC models were treated with drug concentrations at the IC_90_ values derived from 2D cultures. Most agents failed to show comparable efficacy in MTCs relative to 2D cultures; instead, the majority exhibited greater resistance. This indicates that the preserved tumor microenvironment significantly influences drug sensitivity (Figure 3B). We subsequently performed dose-response assays in the MTC model to determine its intrinsic IC_50_ values (Figure S3A). We compared IC_50_ values from MTC and 2D cultures. While some drugs had similar effects in both systems, most of them were less potent in the MTC model. This difference highlights the impact of the microenvironment on drug response (Figure 3C and Figure S3B). The reduced efficacy of several cytotoxic and targeted agents observed in the MTC platform is consistent with extensive evidence that TME drive non-cell-autonomous drug resistance. Doxorubicin, cisplatin, and microtubule-targeting agent show attenuated activity in ECM-rich, hypoxic, or stromal-supported tumors due to limited drug penetration, CAF- and macrophage-mediated survival signaling, and activation of IL-6/STAT3, NF-κB, and AKT pathways 29-32. These mechanisms are almost absent in 2D cultures. CDK4/6 inhibitors such as palbociclib are highly effective in proliferative monolayers but display reduced efficacy in TME-intact systems. This phenomenon is driven by matrix-dependent cell-cycle adaptation and immune-mediated effects. These factors cooperatively suppress T-cell proliferation while preserving cytotoxic function, ultimately limiting overall antitumor efficacy 33, 34. These findings align with prior clinical and preclinical studies: TME-driven, non-cell-autonomous resistance substantially constrains drug efficacy *in vivo*.

We next investigated whether T cells contribute to these differential drug sensitivities. 4T1 breast cancer cells were implanted into immunocompetent BALB/c mice and immunodeficient Nude mice (lacking mature T cells). MTC models derived from these tumors showed that certain drugs, including doxorubicin and epirubicin, were more effective in tissues from immunodeficient mice. Other drugs, such as gemcitabine and paclitaxel, had comparable efficacy regardless of immune status (Figure 3D, E, J, M). These MTC-based findings were consistent with *in vivo* drug responses: doxorubicin and epirubicin treatment resulted in better tumor control in Nude mice than in BALB/c mice, while gemcitabine and paclitaxel showed similar efficacy in both mouse strains (Figure 3F-I, K-L and Figure S3C-D, S4A-D).

To further evaluate whether the MTC platform can predict immune-dependent drug responses beyond a single tumor model, we extended these analyses to an independent EMT6 breast cancer model and treated with same drugs using MTC models from tumors generated in immunocompetent and immunodeficient mice. Consistent with the 4T1 model, doxorubicin and epirubicin again showed greater efficacy in EMT6 tumors derived from immunodeficient mice. In contrast, axitinib and regorafenib, demonstrated enhanced antitumor efficacy in EMT6 and 4T1 MTC models derived from immunocompetent mice, with concordant results observed *in vivo* (Figure S3E, S4E-J). This concordance confirms that the MTC model reliably mirrors *in vivo* drug response patterns.

Given the observed immune-dependent differences in drug efficacy, we further systematically characterized how immune cells modulate drug responses. A library of clinically used drugs was screened using MTC models from 4T1 and EMT6 tumors grown in either immunocompetent or immunodeficient mice. Tumor tissues in MTC were treated with a range of drug concentrations, and viability was assessed by MTT assay after four days (Figure S5A). Analysis of IC_50_ values identified 22 drugs with enhanced efficacy in immunocompetent-derived MTC models and 19 drugs that were more effective in immunodeficient-derived cultures, suggesting that certain agents can activate or require immune responses for full activity, while others may be hampered by the immune microenvironment (Figure 3N and Figure S5B, C). Several agents showing enhanced efficacy in immunocompetent-derived MTCs are known to interact with immune or inflammatory signaling pathways. For example, JAK/STAT inhibitors (*e.g.*, ruxolitinib), multikinase inhibitors (*e.g.*, regorafenib, axitinib and dasatinib), and epigenetic regulators (*e.g.*, vorinostat) have been reported to modulate cytokine signaling, enhance T-cell activation, or reshape immunosuppressive microenvironments 35-42. Conversely, agents exhibiting higher potency in immunodeficient conditions, such as anthracyclines and antimetabolites, including anthracyclines (doxorubicin, epirubicin), hypomethylating agents (azacitidine), and targeted therapies with prominent resistance phenotypes in immune-intact settings (*e.g.*, vemurafenib), are known to be more susceptible to TME-mediated resistance, including stromal support and inflammatory signaling 43-46. These findings provide a mechanistic basis for the differential drug responses observed in the MTC system. Collectively, these results validate the MTC platform as a highly physiologically relevant *ex vivo* system that accurately recapitulates the immune-modulated drug responses observed *in vivo*, providing a powerful tool for predicting drug efficacy and identifying immune-dependent therapeutics.

### MTC model faithfully recapitulates response to immune checkpoint blockade therapy

**Figure 4:**
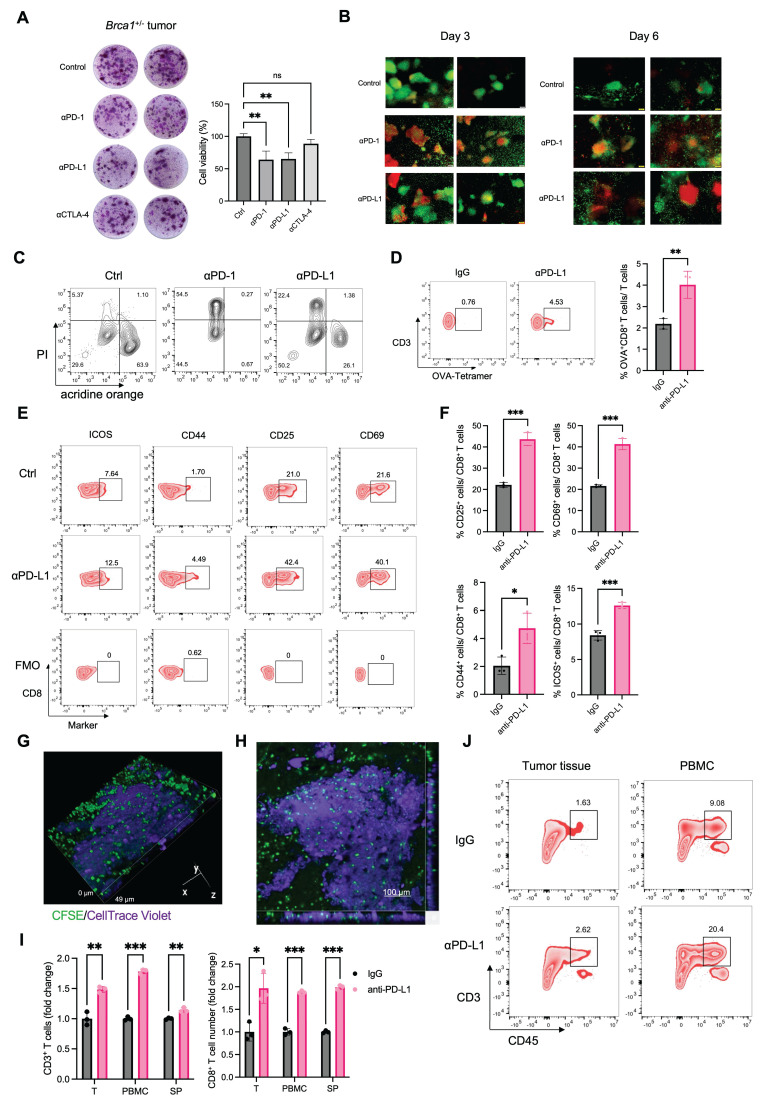
** MTC model faithfully recapitulates response to immune checkpoint blockade therapy.** (A) (Left) Representative images of MTT staining in *Brca1*^+/-^ breast tumor MTC models following treatment with the indicated ICB drugs. (Right) Quantification of cell viability under each condition (n = 3). (B) Representative images of AOPI staining in *Brca1*^+/-^ breast tumor MTC models treated with the indicated ICB drugs at day 3 and day 6 of culture. Two representative fields from the same treatment group are shown. (C) Flow cytometry analysis of viable cells by AOPI staining in *Brca1*^+/-^ MTC models treated with the indicated ICB drugs at day 3. (D) (Left) Flow cytometry analysis of antigen-specific (SIINFEKL-tetramer⁺) CD8⁺ T cell frequency in MC38-OVA tumor-derived MTC models following treatment with control IgG or anti-PD-L1. (Right) Quantification of CD8⁺ OVA⁺ T cells among total CD8⁺ T cells (n = 3). (E) Flow cytometry analysis of T cell activation markers (CD25, CD69, CD44, ICOS) on CD8⁺ T cells in MC38-OVA MTC models after treatment with IgG or anti-PD-L1. FMO, fluorescence minus one control. (F) Quantification of CD25^+^, CD69^+^, CD44^+^ and ICOS^+^ cells among CD8^+^ T cells from (E) (n = 3). (G) Three-dimensional reconstruction of a MTC coculture after 3 days, showing CFSE-labeled splenocytes (green) infiltrating CellTrace Violet-stained tumor tissue (violet). (H) Orthogonal slice views from the 3D image stack in (G). Scale bar is indicated. (I) PBMCs or splenocytes were cocultured with Hepa1-6 tumor tissues in the MTC system. Quantification of fold changes in CD3^+^, CD8^+^ T cell numbers treated with IgG or anti-PD-L1 (n = 3). T, tumor tissues alone; PBMC, MTC co-culture with PBMCs; SP, MTC co-culture with splenocytes. (J) Flow cytometry analysis showing the frequency of CD3⁺ T cells in a human NSCLC sample (NSCLC-4) MTC model, co-cultured with or without autologous PBMCs, following treatment with IgG or anti-PD-L1. Data are presented as mean ± SD; ns, not significant; *p<0.05; **p<0.01; ***p<0.001.

A key advantage of the MTC model over organoid cultures is its preservation of autologous T cell populations. This makes it possible to evaluate ICB drug responses. We then examined whether the MTC system could reliably detect ICB-induced effects. *BRCA1*⁺/⁻ tumor tissues were dissociated and cultured under MTC conditions, and drug responses were evaluated after 7 days. Using MTT viability assay, we found that treatment with either anti-PD-1 or anti-PD-L1 antibodies significantly reduced cell viability. This demonstrates that the MTC platform combined with MTT readout can effectively detect ICB-mediated therapeutic effects (Figure 4A). To further validate these findings, we employed an acridine orange/propidium iodide (AOPI) assay, which allows direct discrimination of live and dead cells. Both confocal microscopy and flow cytometry analyses confirmed that AOPI staining detected clear responses to anti-PD-1 and anti-PD-L1 treatment in *BRCA1*^⁺/⁻^ MTC models (Figure 4B, C and Figure S6A).

We next investigated whether the MTC model could recapitulate antigen-specific T cell responses. MC38 cells expressing ovalbumin (OVA) were implanted into mice, and resulting tumors were cultured in the MTC system. To track antigen-specific CD8⁺ T cells, we used a fluorescently conjugated MHC tetramer specific for the SIINFEKL peptide. Flow cytometry analysis showed a significant increase in OVA-specific CD8⁺ T cells following anti-PD-L1 treatment (Figure 4D). These T cells also exhibited upregulated expression of early activation markers (CD25, CD69) as well as effector and co-stimulatory molecules (CD44, ICOS), as quantified by flow cytometry (Figure 4E, F). These results indicate that the MTC model recapitulates essential features of antigen-specific T cell activation and expansion in response to ICB.

Given previous reports that ICB enhances immune cell infiltration and primes T cells within secondary lymphoid organs, we also assessed immune cell trafficking in the MTC system using confocal microscopy and flow cytometry 47, 48. We first evaluated whether immune cells could infiltrate tumor tissues under MTC conditions. CFSE-labeled immune cells and CellTrace Violet-stained tumor tissues were co-cultured for 3 days, followed by Z-stack confocal imaging and 3D reconstruction. The images revealed substantial infiltration of immune cells into tumor fragments, evidenced by the spatial colocalization of CFSE and CellTrace Violet fluorescence in the core regions of the tissue (Figure 4G, H). We further evaluated whether ICB could enhance this infiltration using a Hepa1-6 murine hepatoma model co-cultured with PBMCs or splenocytes. Anti-PD-L1 treatment significantly increased the infiltration of CD45⁺ immune cells, CD3⁺ T cells, and CD8⁺ T cells, as measured by flow cytometry (Figure 4I and Figure S6B). Similarly, using a human lung cancer specimen co-cultured with autologous PBMCs, anti-PD-L1 treatment enhanced T cell infiltration and improved drug response (Figure 4J and Figure S6C). These data collectively demonstrate that the MTC co-culture system faithfully models ICB-induced immune recruitment in both murine and human tumors.

### High-throughput screening in the MTC model identifies synergistic drug combinations with immune checkpoint blockade

**Figure 5:**
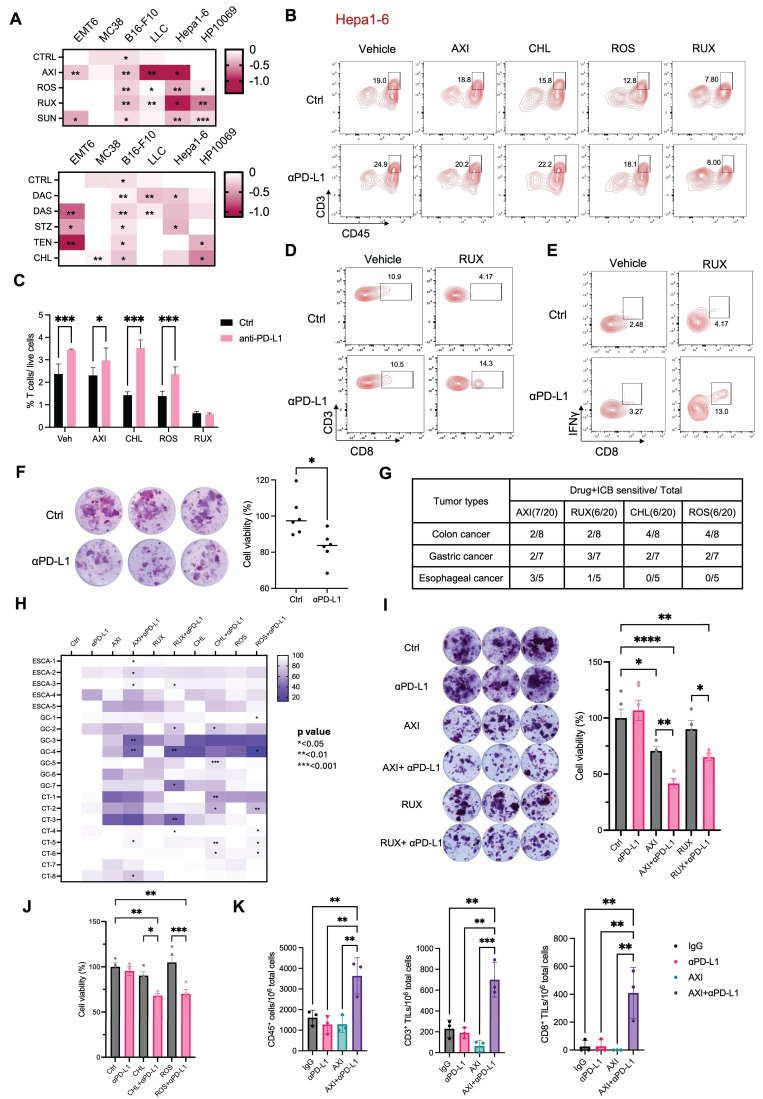
** High-throughput screening in the MTC model identifies synergistic drug combinations with immune checkpoint blockade.** (A) Heatmap of the log fold change in cell viability following treatment with anti-PD-L1 compared to IgG control across tumor MTC models combined with the indicated drugs. Data are presented as mean values. (B) Flow cytometry analysis showing the frequency of CD3⁺ T cells in Hepa1-6 MTC model treated with IgG or anti-PD-L1 combined with the indicated drugs. (C) Quantification of T cell population among live cells in Hepa1-6 MTC model treated with IgG or anti-PD-L1 combined with the indicated drugs (n = 3). (D) Flow cytometry analysis showing the frequency of CD8⁺ T cells in Hepa1-6 MTC model treated with IgG or anti-PD-L1 combined with RUX. (E) Flow cytometry analysis showing the frequency of IFNγ^+^ CD8⁺ T cells in Hepa1-6 MTC model treated with IgG or anti-PD-L1 combined with RUX. (F) (Left) Representative images of MTT assay in a human colon sample treated with IgG or anti-PD-L1. (Right) Corresponding quantification of cell viability under each condition (n = 6). (G) A table summarized the efficacy of IgG or anti-PD-L1 combined with the indicated drugs across various human cancer types. (H) Heatmap of the cell viability following treatment with anti-PD-L1 or IgG in human tumor MTC models combined with the indicated drugs. (I) (Left) Representative images of MTT assay in a human gastric cancer sample treated with IgG or anti-PD-L1 combined with the indicated drugs. (Right) Quantification of cell viability under each condition (n ≥ 4). (J) Quantification of cell viability in a human colon cancer sample treated with IgG or anti-PD-L1 combined with the indicated drugs (n ≥ 3). (K) FACS quantitation of CD45^+^ immune cell, CD3^+^ T cell and CD8^+^ T cell number among 10^6^ live cells in a NSCLC-2 human sample treated with IgG or anti-PD-L1 combined with AXI (n = 3). Data are presented as mean ± SD; *p < 0.05; **p < 0.01; ***p < 0.001; ****p < 0.0001.

To systematically identify agents capable of enhancing ICB efficacy, we conducted a high-throughput drug screen using the MTC platform. Six distinct tumor models—HP10069 (breast cancer), EMT6 (breast cancer), Hepa1-6 (hepatoma), LLC (lung cancer), B16-F10 (melanoma), and MC38 (colon adenocarcinoma)—were screened against a library of 90 drugs (Table S1). Tumor tissues were treated with each drug either alone or in combination with 5 µg/mL anti-PD-L1, and cell viability was assessed by MTT assay after 4 days of culture. A heatmap visualizing the log ratio of viability in combination versus monotherapy groups revealed that a subset of drugs exhibited enhanced cytotoxicity when co-administered with anti-PD-L1, suggesting potential synergistic interaction with ICB (Figure S7A, C), whereas other compounds appeared to attenuate ICB response (Figure S7B, D). Cross-comparison of combination effects across tumor types identified five drugs that synergized with ICB in three different models and four drugs effective in four models (Figure S7C and Figure 5A). Among these nine candidates, axitinib (AXI), sunitinib (SUN), dacomitinib (DAC), and dasatinib (DAS) are tyrosine kinase inhibitors; rosiglitazone (ROS) is a PPAR-γ agonist; ruxolitinib (RUX) is a JAK1/2 inhibitor; and chlorambucil (CHL), streptozotocin (STZ), and teniposide (TEN) are conventional chemotherapeutic agents. Several of these agents are known to synergize with immune checkpoint blockade, based on preclinical or clinical evidence. For instance, axitinib and sunitinib suppress VEGF-driven immunosuppression, normalize tumor vasculature, and reduce MDSCs and regulatory T cells, thereby enhancing effector T-cell infiltration and function and providing a strong mechanistic and clinically validated basis for ICB synergy 35, 36. Dasatinib has been shown to augment cytotoxic lymphocyte activity, including expansion and memory differentiation of γδ T cells, shifting the balance toward immune activation 39, 42. Ruxolitinib enhances ICB by inhibiting JAK1/2-STAT signaling that drives suppressive myeloid cells, thereby reducing MDSCs and restoring antigen-presenting, T-cell–supportive myeloid functions. This myeloid reprogramming increases T-cell and NK-cell activity, sensitizing tumors to PD-1 blockade 49. In contrast, other agents such as chlorambucil, streptozotocin, and teniposide showed limited or less definitive evidence of immunotherapy synergy. This suggest that their effects in the MTC system remains context-dependent or not established.

We next evaluated whether these synergistic drugs modulate T cell activity. Hepa1-6-derived MTC models were treated with AXI, ROS, RUX, or CHL, either alone or in combination with anti-PD-L1 for 4 days. Flow cytometry analysis showed that AXI, ROS, and CHL combined with anti-PD-L1 significantly increased T cell frequencies compared with the respective monotherapies, whereas RUX did not (Figure 5B, C). However, further analysis revealed that RUX in combination with anti-PD-L1 enhanced both the frequency of CD8⁺ T cells and their production of IFNγ, indicating its potential role in promoting cytotoxic T cell function (Figure 5D, E and Figure S8E). Similar evaluations were conducted in the MC38 model. Of the agents tested, only CHL synergized with anti-PD-L1. Consistent with the efficacy data, CHL in combination with anti-PD-L1 significantly augmented T-cell infiltration within MC38-derived MTCs (Figure S8A and S8B). Similarly, in the EMT6 model, where AXI was the only synergistic agent, AXI in combination with anti-PD-L1 specifically increased CD8⁺ T cell frequencies (Figure S8C, D). These findings validate that the synergistic drug candidates identified by screening enhance ICB response through T cell activation.

We further evaluated the translational relevance of these findings using human tumor samples. A cohort of 30 patient-derived specimens—including colon, breast, gastric, ovarian, and esophageal carcinomas—was established within the MTC system (Figure S8F). Only three samples (10%) responded to anti-PD-L1 monotherapy (Figure 5F and Figure S8G). To validate the synergistic efficacy of the selected drugs, we analyzed an additional cohort of 20 patient-derived specimens comprising colon, gastric and esophageal carcinomas. While ICB treatment alone elicited no significant response in these samples, combination therapy markedly enhanced ICB efficacy: AXI increased ICB response rates to 35%, RUX to 30%, CHL to 30%, and ROS to 30% (Figure 5G, H). MTT assay of MTC model confirmed that these combinations enhanced ICB-induced cytotoxicity across human samples (Figure 5I, J and Figure S8H).

To enhance the translational relevance of the patient-derived MTC cohort, we examined available clinical and molecular characteristics and evaluated their concordance with functional ICB responses observed in the MTC system (Table S2). Analyses focused on established biomarkers, including PD-L1 expression and mismatch repair (MMR) status, as well as selected oncogenic features. Consistent with clinical evidence indicating that MMR deficiency (dMMR/MSI-H) is the most reliable predictor of PD-1/PD-L1 blockade in colorectal cancer and a strong predictor of response across several other malignancies 50-52, MTC-predicted ICB responses showed strong concordance (83.3%) with MMR status, with dMMR tumors responding and pMMR tumors remaining non-responsive. In contrast, PD-L1 expression is known to have limited predictive accuracy in esophageal squamous cell carcinoma and gastric cancer and little predictive value in colorectal cancer 53-55. Accordingly, in esophageal and gastric cancers, the concordance (defined as PD-L1 CPS > 10 associated with ICB response and PD-L1 CPS ≤ 10 associated with no response) between PD-L1 expression and MTC-detected ICB response was moderate (54.5%), consistent with the known limited predictive accuracy of PD-L1 alone. Additional molecular features, such as EGFR or HER2 expression and *TP53* mutation status, influenced immune infiltration but were insufficient as standalone predictors of response 56, 57. These results indicate that ICB responses captured by the MTC platform reflect the established strengths and limitations of current clinical biomarkers, while providing an integrated functional readout that incorporates multiple tumor-intrinsic and immune-related factors simultaneously.

Together, these results demonstrate that the MTC platform is useful for high-throughput identification of drugs that potentiate ICB response, thus providing a compelling strategy for overcoming ICB resistance across diverse human cancers.

### Axitinib potentiates ICB efficacy by enhancing antigen presentation and inducing CXCL13-mediated T cell recruitment

**Figure 6:**
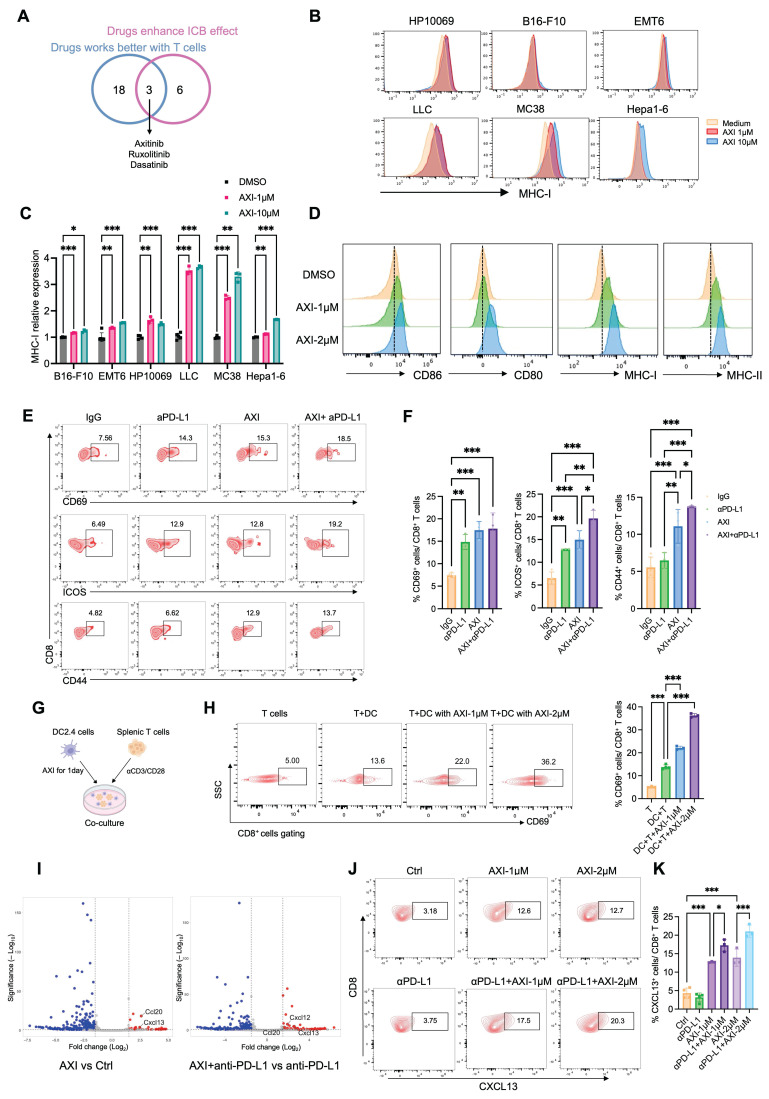
** Axitinib potentiates ICB efficacy by enhancing antigen presentation and inducing CXCL13-mediated T cell recruitment.** (A) Venn diagram identifies overlapping drugs that exhibit both superior efficacy in immunocompetent-derived MTC models and synergistic activity with ICB. (B) Flow cytometry analysis of MHC-I expression in various tumor cell lines treated with 1 or 10 μM AXI. (C) Quantification of MHC-I expression in various cell lines treated with AXI (n ≥ 3). (D) Flow cytometry analysis of CD80, CD86, MHC-I and MHC-II expression in DC2.4 cells treated with 1 or 2 μM AXI. (E) Profiling of T cell activation marker (CD69, CD44, ICOS) on CD8^+^ T cells after treatment with anti-PD-L1 alone or combined with AXI. (F) Quantification of CD69^+^, CD44^+^ and ICOS^+^ cell proportion among CD8^+^ T cells (n ≥ 3). (G) Schematic diagram illustrating procedure for co-culturing AXI-pretreated DC2.4 cells with splenic T cells. Created in BioRender. Von, Y. (2026) https://BioRender.com/or51j3k. (H) (Left) Flow cytometry analysis of CD69 expression on CD8^+^ splenic T cells after co-culturing with DMSO or AXI-pretreated DC2.4 cells. (Right) Quantification of CD69^+^ cell proportion among CD8^+^ T cells (n ≥ 3). (I) Volcano plot from bulk RNA-seq analysis of Hepa1-6 MTC models treated with anti-PD-L1 alone or combined with AXI. (J) Flow cytometry analysis of CXCL13^+^ cell proportion among CD8^+^ T cells after treatment with anti-PD-L1 alone or combined with AXI. (K) Quantification of CXCL13^+^-producing CD8^+^ T cells after treatment with anti-PD-L1 alone or combined with AXI (n ≥ 3). Data are presented as mean ± SD; ns, not significant; *p < 0.05; **p < 0.01; ***p < 0.001.

We then screened for agents that both improve ICB response and exhibit enhanced efficacy in immunocompetent settings (Figure S5B and Figure 5A). We identified axitinib, ruxolitinib, and dasatinib, each of which both enhances ICB effects and activates T cells (Figure 6A). The JAK1/2 inhibitor RUX is already in clinical trials in combination with ICB for non-small cell lung cancer and Hodgkin lymphoma, which lines up with what we observed 49, 58. DAS has also been examined in combination with ICB in acute lymphoblastic leukemia 59. AXI, a VEGFR inhibitor, is FDA-approved in combination with ICB for renal cell carcinoma, but it is not widely used in other solid tumors. Our data indicate that AXI enhances ICB response in multiple mouse and human cancer types, suggesting its clinical utility could extend beyond the current indication.

To elucidate the mechanisms of AXI’s synergy with ICB beyond VEGFR inhibition, we first assessed immune cell changes in human lung cancer MTC models. Combination treatment significantly increased CD45⁺ immune cells, CD3⁺ T cells, and CD8⁺ T cells, indicating enhanced T cell activation (Figure 5K). Since T cell activation depends on antigen presentation, we evaluated MHC-I expression on tumor cells. We found that AXI upregulated MHC-I across multiple tumor types (Figure 6B, C). We next examined dendritic cell (DC) function using the DC2.4 cell line. AXI treatment elevated expression of antigen presentation molecules (MHC-I, MHC-II) and co-stimulatory markers (CD80, CD86), indicating enhanced DC maturation and antigen-presenting capacity (Figure 6D and Figure S9A). To track antigen-specific CD8^+^ T cell responses, we examined T cell activation marker. Flow cytometry analysis of T cell activation markers revealed that AXI alone, and more strongly in combination with anti-PD-L1, increased levels of the early activation marker CD69 as well as the activation and co-stimulatory molecules CD44 and ICOS, indicative of a high-quality, sustained T cell response (Figure 6E, F). In a DC-T cell co-culture system, AXI-pretreated DC2.4 cells markedly increased the frequency of CD69⁺ CD8⁺ T cells, confirming that AXI potentiates DC-mediated T cell activation (Figure 6G, H).

To further unravel the molecular mechanisms of AXI-induced T cell activation, we performed bulk RNA-seq and identified *Cxcl13* as a key markedly upregulated gene in both AXI and AXI+anti-PD-L1 treated groups (Figure 6I). To extend this analysis beyond individual differentially expressed genes, we conducted gene set enrichment analysis (GSEA), which revealed significant enrichment of immune-related signaling pathways in AXI-treated samples, including interferon-α response, interferon-γ response, IL-6–JAK–STAT3 signaling, and IL-2–STAT5 signaling. These pathways are closely associated with immune activation, T-cell function, and cytokine-driven immune responses (Figure S9D). Consistent with these pathway-level changes, analysis of human cancer databases revealed that high CXCL13 expression correlates with prolonged relapse-free and overall survival across multiple tumor types, supporting its role as a favorable biomarker for immunotherapy response (Figure S9B, C). We then assessed cellular sources of CXCL13 in the Hepa1-6 MTC model and found that the combination therapy increased CXCL13 expression in T cells and DCs, with the most pronounced elevation in CD8⁺ T cells (Figure 6J, K and Figure S9E, F).

To assess the functional contribution of CXCL13 signaling to AXI-associated T cell activation, we performed CXCL13 neutralization experiments in the EMT6 MTC model. Treatment with a CXCL13-blocking antibody partially attenuated AXI- and AXI + anti-PD-L1–induced tumor cell death and significantly reduced CD8⁺ T-cell frequency and activation, as indicated by decreased expression of CD69 and CD44 (Figure S9G-K). These findings indicate that CXCL13 plays a functional role in mediating AXI-associated enhancement of T-cell activation in the tumor context. Thus, beyond its known anti-angiogenic action, AXI enhances ICB efficacy in part by boosting antigen presentation in tumor and dendritic cells and triggering a CXCL13-mediated T cell recruitment and activation program, suggesting a multifactorial immunomodulatory mechanism across diverse cancers.

## Discussion

Our study introduces the miniaturized-tumor culture platform, a novel and optimized *ex vivo* culture system that uniquely maintains a functional tumor immune microenvironment, including autologous T cells, for high-throughput drug screening. We optimized the MTC platform to produce an *ex vivo* model that preserves the native human tumor immune microenvironment and is scalable. We found that larger tissue fragments (100–500 μm) are superior in maintaining immune cell populations and overall viability. Based on that, we developed a system suitable for high-throughput drug screening. A key discovery was the identification of IL-7, with or without IL-2, allows the long-term maintenance of functional T cell populations for over two weeks in cultures derived from diverse human cancers. This optimization successfully addresses a major limitation of previous *ex vivo* models—the rapid loss of immune components. As a result, the MTC is a powerful tool for prolonged immuno-oncology studies and personalized therapeutic screening. Using MTC, we identified several drugs that synergize with ICB, especially the VEGFR inhibitor axitinib, which significantly enhanced ICB efficacy in human tumor samples. Mechanistic investigations revealed that AXI potentiates ICB response not only through its anti-angiogenic effects but also by enhancing antigen presentation in tumor and dendritic cells. It also induced CXCL13-associated T cell responses, shows it acts as a contributory immunomodulatory role rather than a single dominant mechanism. Our findings suggest MTC is a powerful tool for accelerating immunotherapy discovery and provide a reason for clinical testing of AXI-ICB combinations in a broad range of cancers.

The MTC platform represents a functionally validated evolution of existing organotypic tumor culture systems. Classical preclinical models, such as 2D cell lines and 3D organoids, lack autologous immune compartments. TME-preserving platforms, including tumor slices, tumor fragment culture and air–liquid interface systems, can partially retain native tissue architecture and immune cells. However, these platforms are commonly limited by a rapid decline in immune cell numbers, restricted experimental flexibility, and poor scalability 17-19, 22. They also struggle to preserve functional autologous T cells, support immune cell infiltration and trafficking, or integrate with systematic high-throughput drug screening.

Through systematic optimization of tumor fragment size and cytokine support, especially IL-7, the MTC platform maintains viable and functional autologous T cells for therapeutically relevant durations over two weeks across diverse murine and human tumor types. In contrast, previous organotypic studies using smaller fragments (40–100 μm) reported only transient immune retention with progressive T-cell functional decline 20, 21. Beyond immune preservation, the miniaturized and standardized architecture of MTCs fits directly into multi-well formats with quantitative readouts. This enables scalable, parallel screening of drug combinations, which is not practical for most existing TME-preserving systems. Importantly, MTC models also recapitulate immune-dependent drug responses observed *in vivo*, including differential efficacy in immunocompetent versus immunodeficient mice. This allows for direct quantitative comparison, that has rarely been demonstrated in prior organotypic platforms. Finally, the MTC system can be used to study mechanisms of immune-dependent drug synergies that require coordinated activity of different immune cell types. For example, axitinib promotes antigen presentation and CXCL13-associated recruitment of T-cells, including CD8⁺ T cells, CD4⁺ T cells, and dendritic cells.

Recent progress in droplet-based microfluidic systems, such as MicroOrganoSphere (MOS), provide an alternative approach for functional drug testing especially for limited patient-derived material 60. These systems convert dissociated tumor and stromal cells into uniform micro-spheroids, enabling standardized high-throughput analysis. However, MOS relies on enzymatic dissociation, which disrupts native spatial organization and pre-existing tumor–immune interactions. The MTC platform, by contrast, uses intact tumor fragments, preserving endogenous tissue architecture and spatial features of the tumor immune microenvironment. While MOS is well suited for short-term, rapid clinical testing, the MTC platform supports prolonged immune maintenance and enables analysis of delayed responses and mechanistic immune interactions. In addition, MTCs do not rely on specialized microfluidic infrastructure; they use standard laboratory formats instead, which improves both accessibility and scalability. Together, these features make MTC platform a scalable, immune-preserving, and mechanistically informative extension of existing *ex vivo* tumor culture models, thereby addressing key unmet needs in immuno-oncology research and drug discovery.

IL-7 supplementation was incorporated into the MTC platform to prevent the rapid loss of endogenous T cells, which usually occurs in *ex vivo* tumor cultures. IL-7 is well established as a key regulator of T-cell survival through induction of anti-apoptotic pathways, such as Bcl-2 family proteins 61, 62. While prolonged IL-7 exposure has been reported to alter T-cell dynamics in vitro, these observations are largely derived from naïve CD8⁺ T cells. Tumor-infiltrating lymphocytes (TILs) in our system are predominantly antigen-experienced, differentiated, or partially exhausted T cells. These cells are known to respond differently to cytokine signaling and are less prone to IL-7–induced activation-associated cell death 61. What’s more, IL-7 signaling is largely restricted to immune cells, owing to the limited expression of IL-7 receptors on tumor cells. Although IL-7 signals through the JAK/STAT pathway, this signaling in tumor cells is also intrinsically active. To directly evaluate potential confounding effects, we performed comparative analyses of ICB, axitinib, and ruxolitinib under different IL-7 conditions, including reduced or absent IL-7. Across these conditions, drug response profiles remained consistent (Figure S2L–N), indicating that IL-7 supplementation does not substantially alter treatment sensitivity or artificially enhance pathway-specific responses. Taken together, these findings suggest that, under the conditions used, IL-7 mainly functions to preserve T-cell viability and maintain the integrity of tumor–immune interactions in the MTC system.

The MTC platform combines an intact tumor immune microenvironment within a standardized, scalable *ex vivo* system. This establishes a robust and physiologically relevant model for high-throughput drug screening. Its compatibility with multi-well plate formats and endpoint assays such as MTT enables reliable, parallel evaluation of numerous drug conditions with minimal well-to-well variability. The platform demonstrates high predictive validity, accurately recapitulating immune-dependent drug responses—as evidenced by the differential efficacy of axitinib and regorafenib across immunocompetent and immunodeficient contexts. More importantly, the high-throughput screen of 90 drugs identified several novel synergists with anti-PD-L1 therapy. The translational relevance of these findings was strongly supported by testing in a cohort of 20 human patient-derived MTC models, where AXI, for instance, increased the response rate to ICB from 0% to 35%. This demonstrates MTC's powerful utility in discovering and prioritizing combination immunotherapies with high clinical translation potential.

Our findings provide strong evidence for expanding the clinical investigation of these drug combinations. RUX, a JAK1/2 inhibitor, plays a dual role in cancer immunity. While JAK-STAT signaling is crucial for cancer cell proliferation in certain malignancies, its inhibition can also reshape the tumor microenvironment by reducing immunosuppressive cytokines and enhancing T cell function. This dual mechanism may explain why RUX synergizes with ICB in our MTC model. This possibility is further supported by ongoing clinical trials evaluating JAK inhibitors in combination with ICB in Hodgkin lymphoma and non-small cell lung cancer 49, 58, where they aim to overcome cytokine-mediated resistance mechanisms. Dasatinib, a multi-kinase inhibitor of BCR-ABL and SRC family kinases, demonstrates immunomodulatory activity across multiple cancer types. Clinical studies in chronic myeloid leukemia (CML) show that DAS treatment reduces immunosuppressive monocytic myeloid-derived suppressor cells (M-MDSCs). This reduction correlates with Major Molecular Response (MMR) attainment. These findings suggest that DAS may help alleviate MDSC-mediated immunosuppression 63. Supporting this, a preclinical study showed that DAS combined with anti-PD-1 triggers strong antitumor effects in Philadelphia chromosome-positive ALL, leading to smaller tumors and longer survival 59. Beyond hematologic malignancies, DAS shows promise in solid tumors as well. Our MTC results are consistent with a study in small cell lung cancer (SCLC), where combining DAS with anti-PD-1/anti-CTLA-4 produced better outcomes than ICB alone. Mechanistically, DAS reshapes the tumor immune microenvironment by enhancing infiltration of CD4⁺ T cells, CD8⁺ T cells, NK cells, and M1-like macrophages, while decreasing regulatory T cells and M2-like macrophages. DAS also induces CCL5 production in NK cells—a cytokine essential for the antitumor response. This indicates a novel immune-mediated mechanism of DAS in solid tumors 42. Altogether, these findings highlight DAS’s capacity to reverse key mechanisms of immune suppression across diverse cancers, offering a strong rationale for its broader clinical application in combination with immunotherapy.

Notably, our study provides novel mechanistic insights into AXI's immunomodulatory properties beyond its known anti-angiogenic role via VEGFR inhibition. The AXI-ICB combination is already FDA-approved for renal cell carcinoma, but its mechanisms in other cancers remains poorly understood. We found that AXI synergizes with ICB through two distinct mechanisms. First, it enhances antigen presentation by upregulating MHC-I on tumor cells and driving dendritic cell maturation (increased MHC-I/II, CD80, CD86). Second, AXI induces the chemokine CXCL13, which we identified as a key event in this synergy. CXCL13 is an important immune mediator produced by CD103⁺ CD8⁺ T cells upon TGF-ß stimulation; it recruits B cells, drives formation of tertiary lymphoid structures (TLS) and sustains antitumor immunity 64. Across multiple cancer types, high CXCL13 expression correlates with improved patient survival and enhanced responses to PD-1/PD-L1 blockade 65, 66. Mechanistically, CXCL13 contributes to an immunoactive tumor microenvironment by promoting T cell activation and reducing exhaustion, an effect seen in both CAR T-cell models and clinical cohorts 67. What's more, CXCL13-driven TLS formation is associated with better outcomes and more durable ICB responses 68. In line with these observations, recombinant CXCL13 synergized with ICB in our system, reinforcing its role in enhancing antitumor immunity. Together, these results suggest that AXI enhances ICB efficacy through two complementary mechanisms-improved antigen presentation and CXCL13 induction-that create a favorable immune microenvironment promoting T cell recruitment, activation, and TLS development. This provides a promising strategy to overcome ICB resistance. These mechanisms support extending AXI-ICB trials to additional solid tumors, which may help overcome primary ICB resistance. This combination is already being tested in thymic carcinoma 69, hepatocellular carcinoma 70, and gastrointestinal stromal tumors 71. Our findings suggest that CXCL13 expression may serve as a potential predictive biomarker for patient selection.

Our findings carry meaningful clinical implications. The MTC platform could be developed into a personalized medicine tool, allowing patient responses to ICB combinations to be pre-tested *ex vivo* and guiding therapeutic decisions accordingly. The identified drug candidates, especially AXI, provide a strong rationale for clinical trials exploring their combination with ICB in solid tumors beyond their current indications, especially in ICB-resistant cases. Future work will focus on validating these combinations in larger patient cohorts using the MTC platform, as well as on identifying the upstream signaling through which AXI modulates CXCL13 expression.

While the MTC platform effectively preserves functional T-cell populations, it faces limitations in representing and maintaining stromal and myeloid cells over the long term. During early culture, we detected multiple stromal and immune cell types, including myofibroblastic cancer-associated fibroblasts (α-SMA⁺), tumor-associated macrophages (CD11b⁺/F4/80⁺), B cells, NK cells, and dendritic cells. However, compared with primary tumor tissue, macrophages and dendritic cells gradually declined over time. Since these stromal and myeloid components play a critical role in regulating T-cell phenotypes and tumor–immune interactions, this reduction may limit the platform’s ability to fully model certain aspects of the tumor microenvironment. We also acknowledge that spatial heterogeneity cannot be fully eliminated in *ex vivo* systems. Therefore, the MTC platform captures representative but not spatially resolved tumor heterogeneity. In addition, progressive changes in tissue integrity during *ex vivo* culture do not allow long-term passaging or serial expansion of MTC, which may limit its application in extended or iterative experimental studies. Future optimization will focus on improved preservation of these cell populations and the development of strategies to support longer-term culture, which would enhance the modeling of stromal–immune interactions.

## Conclusions

The MTC platform effectively narrows the gap between conventional preclinical models and clinical reality by its better maintenance of a functional TME. Using this system, we have not only validated its predictive power but also uncovered a novel dual mechanism by which AXI synergizes with ICB, involving enhanced antigen presentation and a CXCL13-mediated T cell response. This study provides both a powerful platform and new strategies for advancing combination cancer immunotherapy.

## Supplementary Material

Supplementary figures and tables.

## Data Availability

The data that support the findings of this study are available upon reasonable request.

## Abbreviations

MTC: miniaturized-tumor culture
TME: tumor microenvironment
ICB: immune checkpoint blockade
TIME: tumor immune microenvironment
PBMCs: peripheral blood mononuclear cells
AXI: axitinib
NAMs: New Approach Methodologies
RUX: ruxolitinib
CHL: chlorambucil
ROS: rosiglitazone
PI: propidium iodide
DEGs: differentially expressed genes
SD: standard deviation
ALI: air-liquid interface
SPF: specific pathogen-free
s.c.: subcutaneous
i.p.: intraperitoneal
IC_50_: half-maximal inhibitory concentration
AOPI: acridine orange/propidium iodide
OVA: ovalbumin
GSEA: gene set enrichment analysis
MOS: MicroOrganoSphere
CML: chronic myeloid leukemia
M-MDSCs: monocytic myeloid-derived suppressor cells
MMR: Mismatch Repair
SCLC: small cell lung cancer
TLS: tertiary lymphoid structures
