# Supplementary material for: A miniaturized-tumor culture platform for developing anti-tumor immunotherapies
Source: Theranostics. 2026 Jul 13;16(14):8129–50. doi: 10.7150/thno.132113 (PMC13440502; doi:10.7150/thno.132113)
Supplement: Supplementary file 1 [file thnov16p8129s1.pdf]

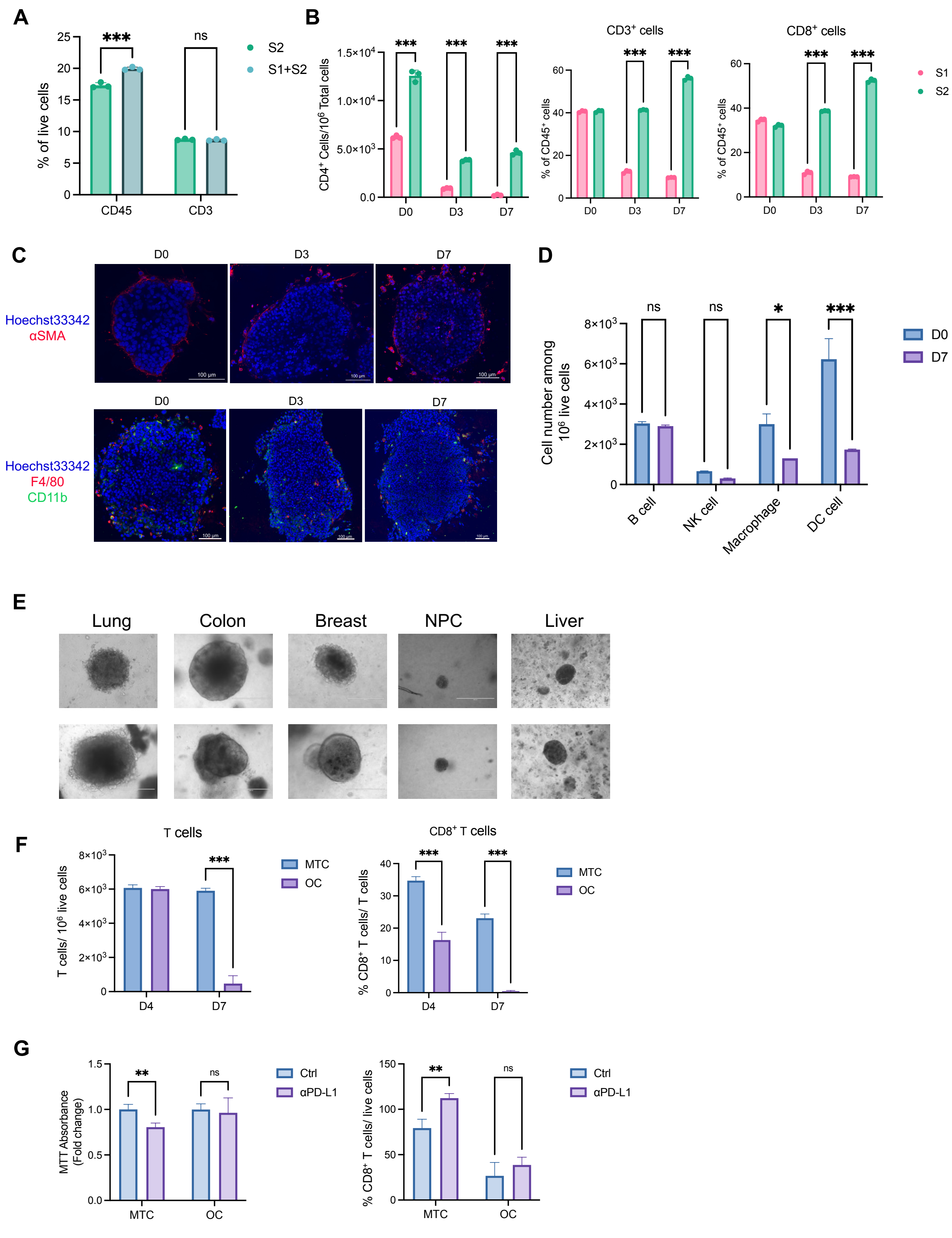

Supplementary Figure 1. Establishment and optimization of the MTC model.

(A) FACS quantification of $CD45^+$ immune cells and $CD3^+$ T cells among live cells in differently sized MTC models from Hepa1-6 tumors at day 3 (n = 3). (B) FACS quantification of $CD4^+$ T cell number among 10^6 live cells, percentage of $CD3^+$ T cells and $CD8^+$ T cells among immune cells in differently sized MTC models from Hepa1-6 tumors at day 3 and day 7 (n = 3). (C) Representative immunofluorescence images of MTC tissues showing myofibroblastic cancer-associated fibroblasts (α-SMA^+, red) and tumor-associated macrophages ($CD11b^+$/$F4/80^+$, green/red) after 3 or 7 days of culture. (D) FACS quantification of B cell, NK cell, macrophage and dendritic cell (DC) number among 10^6 live cells, in MTC models from Hepa1-6 tumors at day 0 and day 7 (n = 3). (E) Representative phase-contrast images of patient tumor fragments among different tumor types cultured in the MTC system. (F) FACS quantification of $CD3^+$ T cell number among 10^6 live cells, percentage of $CD8^+$ T cells among T cells in MTC and organoid cultures (OC) derived from 4T1 tumors at day 4 and day 7 (n = 3). (G) Relative MTT absorbance (n = 4) and FACS quantification of $CD8^+$ T cell number among 10^6 live cells (n = 3) of samples treated with anti-PD-L1 among MTC and organoid cultures derived from 4T1 tumors at day 4. Data are presented as mean ± SD; ns, not significant; *$p<0.05$; **$p<0.01$; ***$p<0.001$.

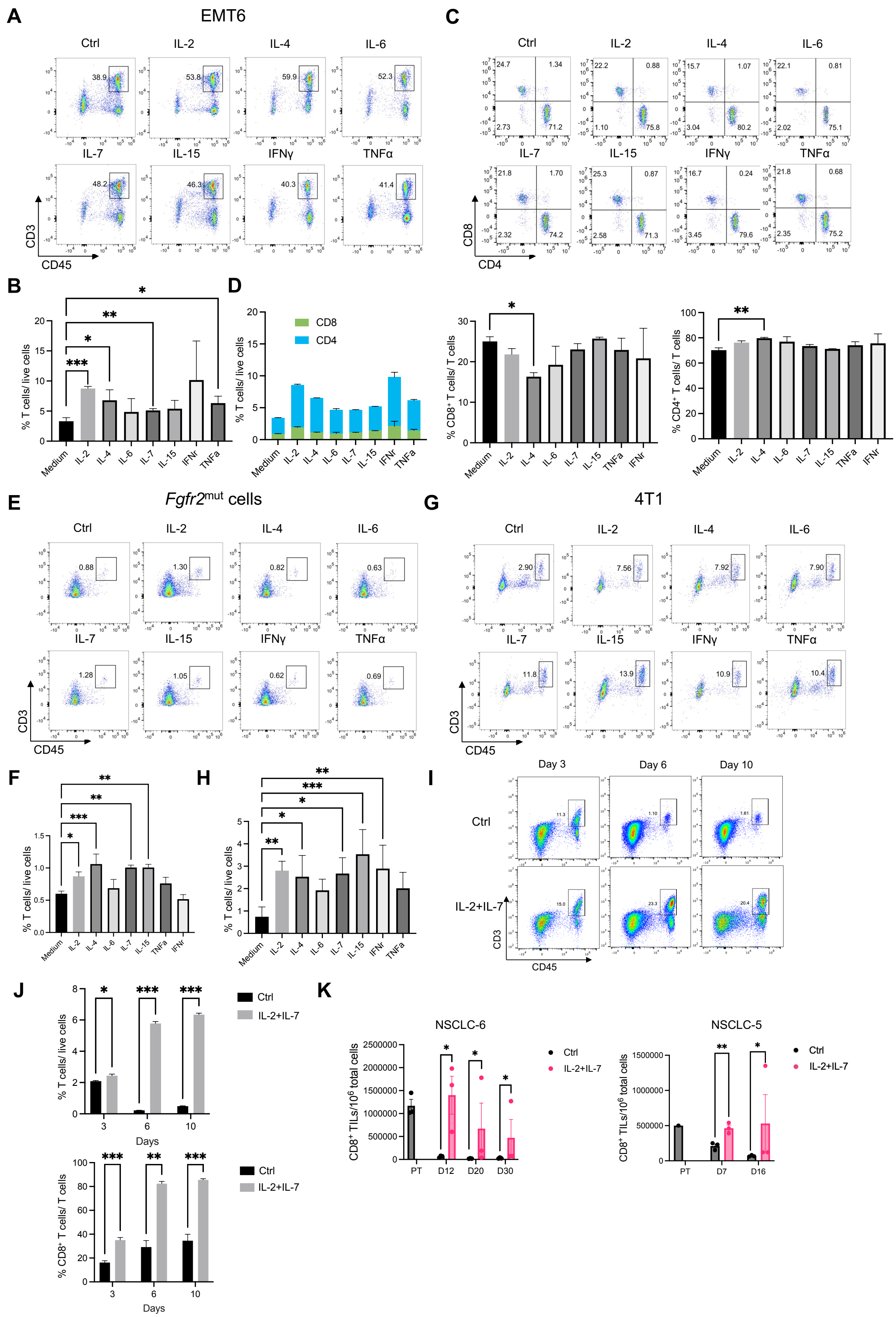

A
EMT6
Ctrl
IL-2
IL-4
IL-6
IL-7
IL-15
IFNγ
TNFα
CD3
CD45
C
CD8
CD4
B
% T cells/ live cells
D
CD8
CD4
% CD8+ T cells/ T cells
% CD4+ T cells/ T cells
Medium
IFNr
TNFa
E
Fgfr2mut cells
G
4T1
F
H
I
Day 3
Day 6
Day 10
IL-2+IL-7
J
Days
K
NSCLC-6
NSCLC-5
CD8+ TILs/10^6 total cells
PT
D12
D20
D30
D7
D16

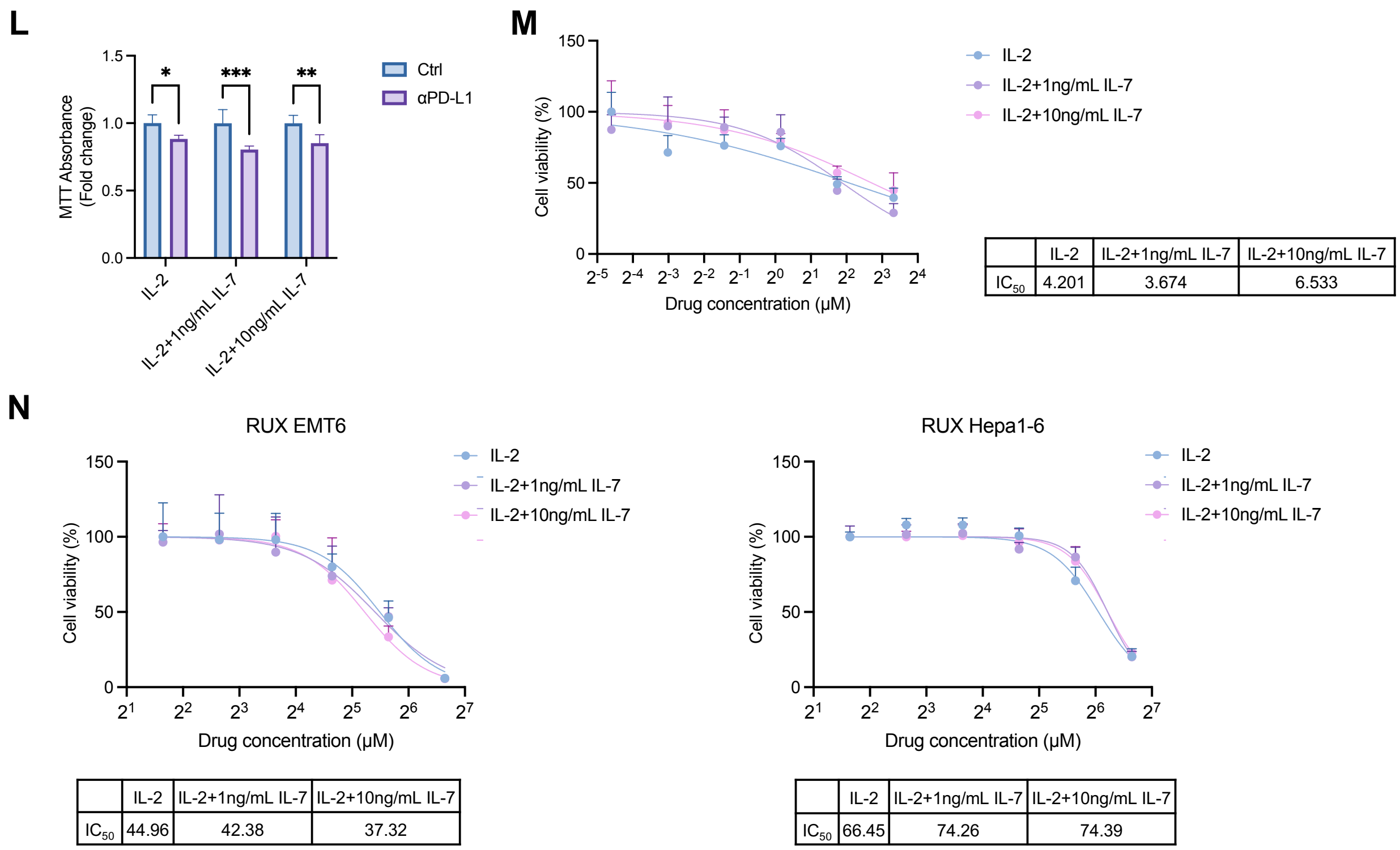

Supplementary Figure 2. IL-7 facilitates the long-term maintenance of T cell populations in MTC model.

(A) Representative flow cytometry analysis showing the proportion of T cells (gated on SSC-low, viable cells) in EMT6 MTC models treated with the indicated cytokines for 3 days. (B) Quantification of T cell proportion among live cells in EMT6 MTC models treated with different cytokines for 3 days ($n \geq 3$). (C) Flow cytometry analysis of $CD8^+$ T cell and $CD4^+$ T cell subsets among $CD3^+$ T cell population in EMT6 MTC models treated with the indicated cytokines for 3 days. (D) Statistical analysis of $CD8^+$ and $CD4^+$ T cell proportion among $CD3^+$ T cells or live cells in EMT6 MTC models treated with different cytokines for 3 days ($n \geq 3$). (E) Representative flow cytometry analysis showing the proportion of T cells (gated on SSC-low, viable cells) in *Fgfr2*^{mut} MTC models treated with the indicated cytokines for 3 days. (F) Quantification of T cell proportion among live cells in *Fgfr2*^{mut} MTC models treated with different cytokines for 3 days ($n = 3$). (G) Representative flow cytometry analysis showing the proportion of T cells (gated on SSC-low, viable cells) in 4T1 MTC models treated with the indicated cytokines for 3 days. (H) Quantification of T cell proportion among live cells in 4T1 MTC models treated with different cytokines for 3 days ($n = 3$). (I) Flow cytometry analysis of T cell proportion (gated on SSC-low, viable cells) in

MC38 MTC models treated with IL-2 combined with IL-7 for 3, 6 and 10 days. (J) Statistical analysis of $CD8^+$ T cell proportion among T cells and $CD3^+$ T cell proportion among live cells in MC38 MTC models treated with IL-2 combined with IL-7 for 3, 6 and 10 days ($n \geq 3$). (K) FACS quantitation of $CD8^+$ T cell number among 10^6 live cells in the NSCLC-5 and NSCLC-6 human samples. MTC models were cultured with or without IL-2 and IL-7 ($n = 3$). (L) Relative MTT absorbance of samples treated with or without anti-PD-L1 among MTC model derived from EMT6 tumors at day 4, with media supplemented with IL-2 alone, IL-2 plus 1 ng/mL IL-7 or IL-2 plus 10 ng/mL IL-7 ($n \geq 3$). (M) Dose-response curves of cell viability in EMT6 MTC models treated with AXI, with media supplemented with IL-2 alone, IL-2 plus 1 ng/mL IL-7 or IL-2 plus 10 ng/mL IL-7 ($n \geq 3$). IC_{50} values are indicated. (N) Dose-response curves of cell viability in EMT6 and Hepa1-6 MTC models treated with RUX, with media supplemented with IL-2 alone, IL-2 plus 1 ng/mL IL-7 or IL-2 plus 10 ng/mL IL-7 ($n \geq 3$). IC_{50} values are indicated. Data are presented as mean ± SD; *p<0.05; **p<0.01; ***p<0.001.

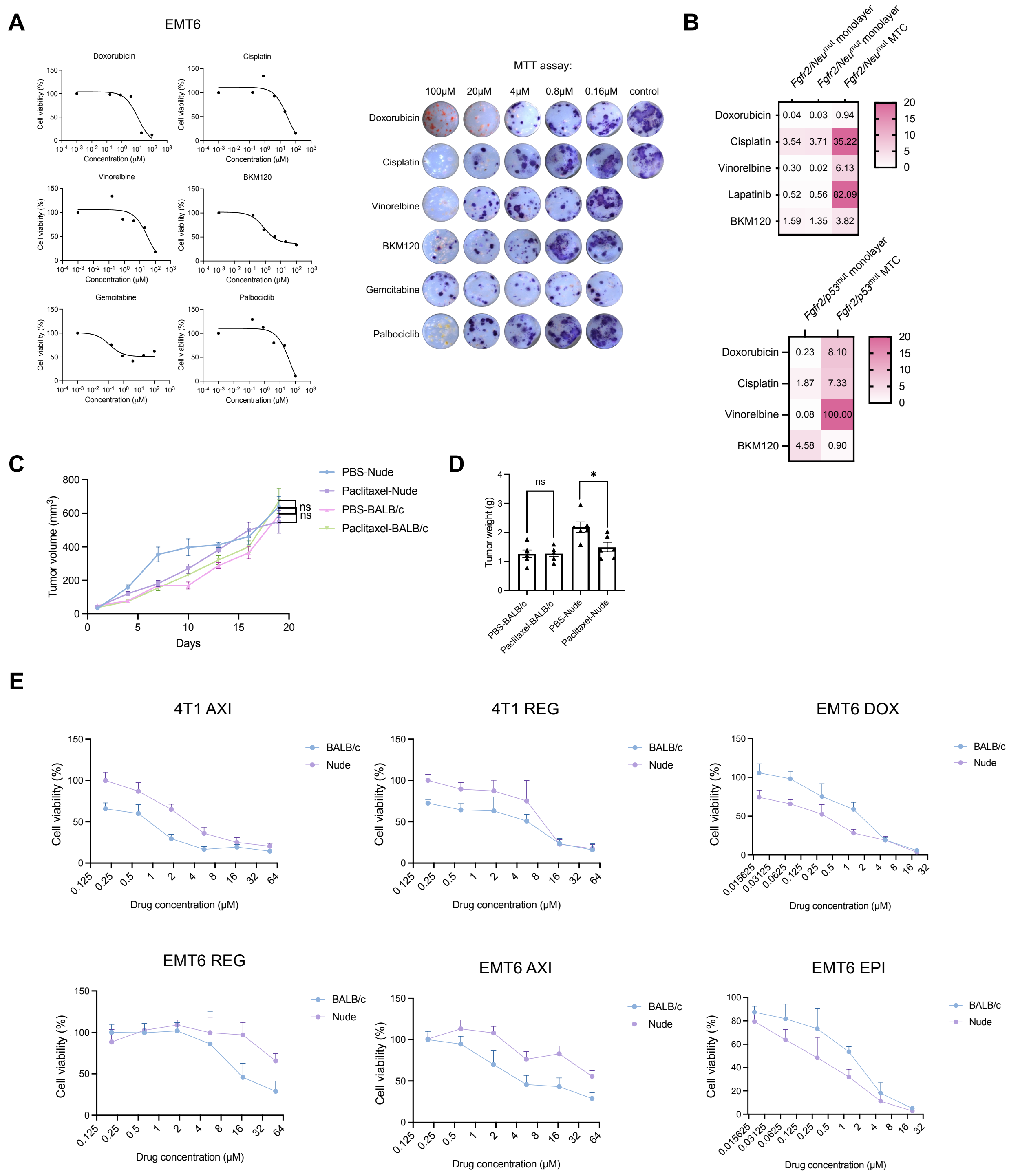

Supplementary Figure 3. MTC model recapitulates *in vivo*-like drug response profiles.

(A) Dose-response curves and representative images of MTT staining in EMT6 breast tumor MTC models derived from immunocompetent (BALB/c) mice after treatment with various drug concentrations. (B) Comparison of drug IC_{50} values between 2D cultures and MTC models. (C) Tumor growth curves of 4T1 cells implanted in Nude mice or BALB/c mice and treated with

paclitaxel (n = 6). Data are presented as mean ± SEM. (D) Comparison of final tumor weights in 4T1-bearing Nude or BALB/c mice treated with paclitaxel (n = 6). Data are presented as mean ± SEM; ns, not significant; *p<0.05. (E) Dose-response curves of cell viability in EMT6 and 4T1 MTC models derived from immunocompetent (BALB/c) mice or immunodeficient (Nude) mice treated with indicated drugs (n = 6). Data are presented as mean ± SD.

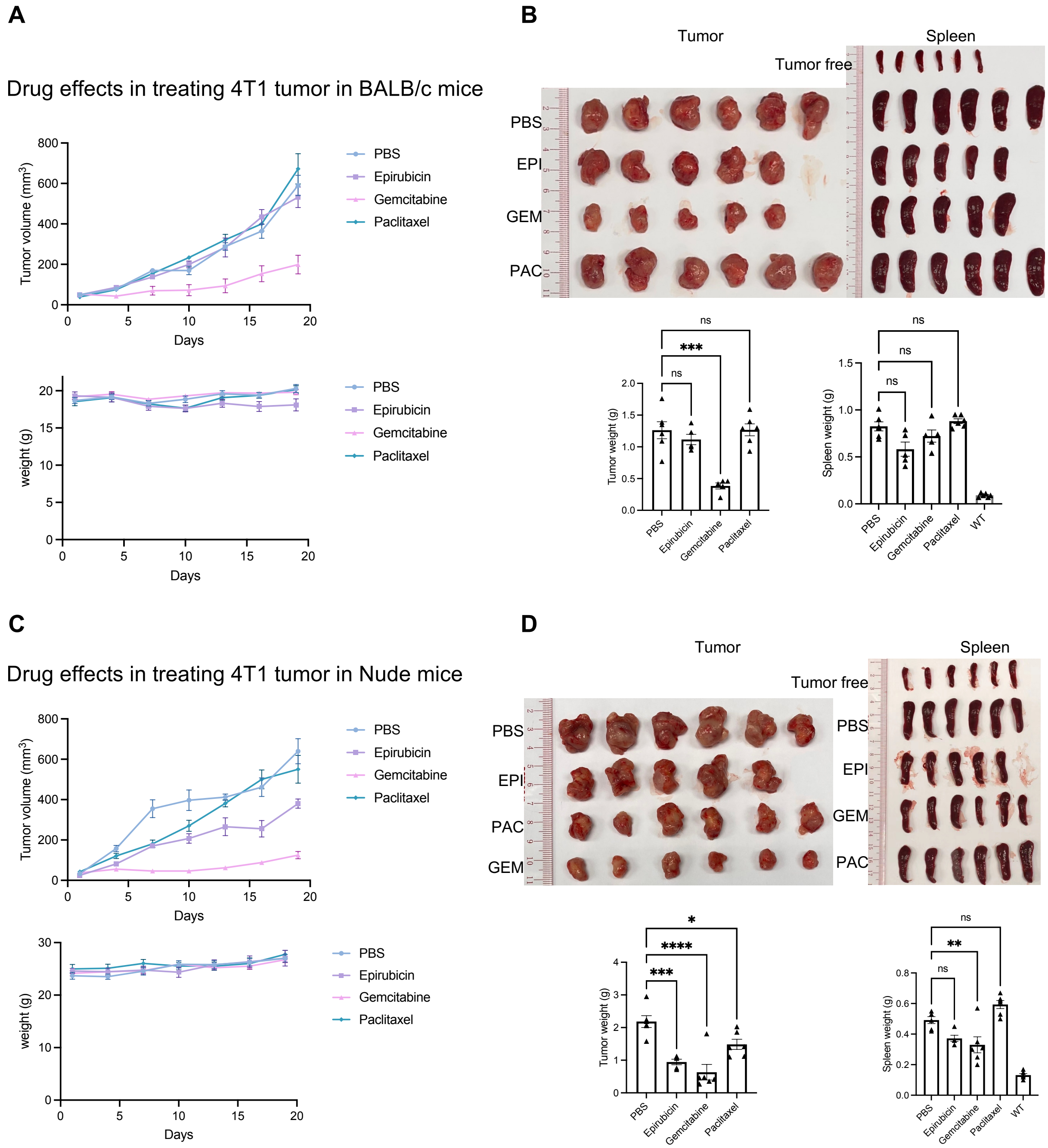

A
Drug effects in treating 4T1 tumor in BALB/c mice
Tumor volume (mm3)
Days
weight (g)
PBS
Epirubicin
Gemcitabine
Paclitaxel
B
Tumor
Spleen
Tumor free
PBS
EPI
GEM
PAC
Tumor weight (g)
Spleen weight (g)
ns
***
WT
C
Drug effects in treating 4T1 tumor in Nude mice
D
*
****
**

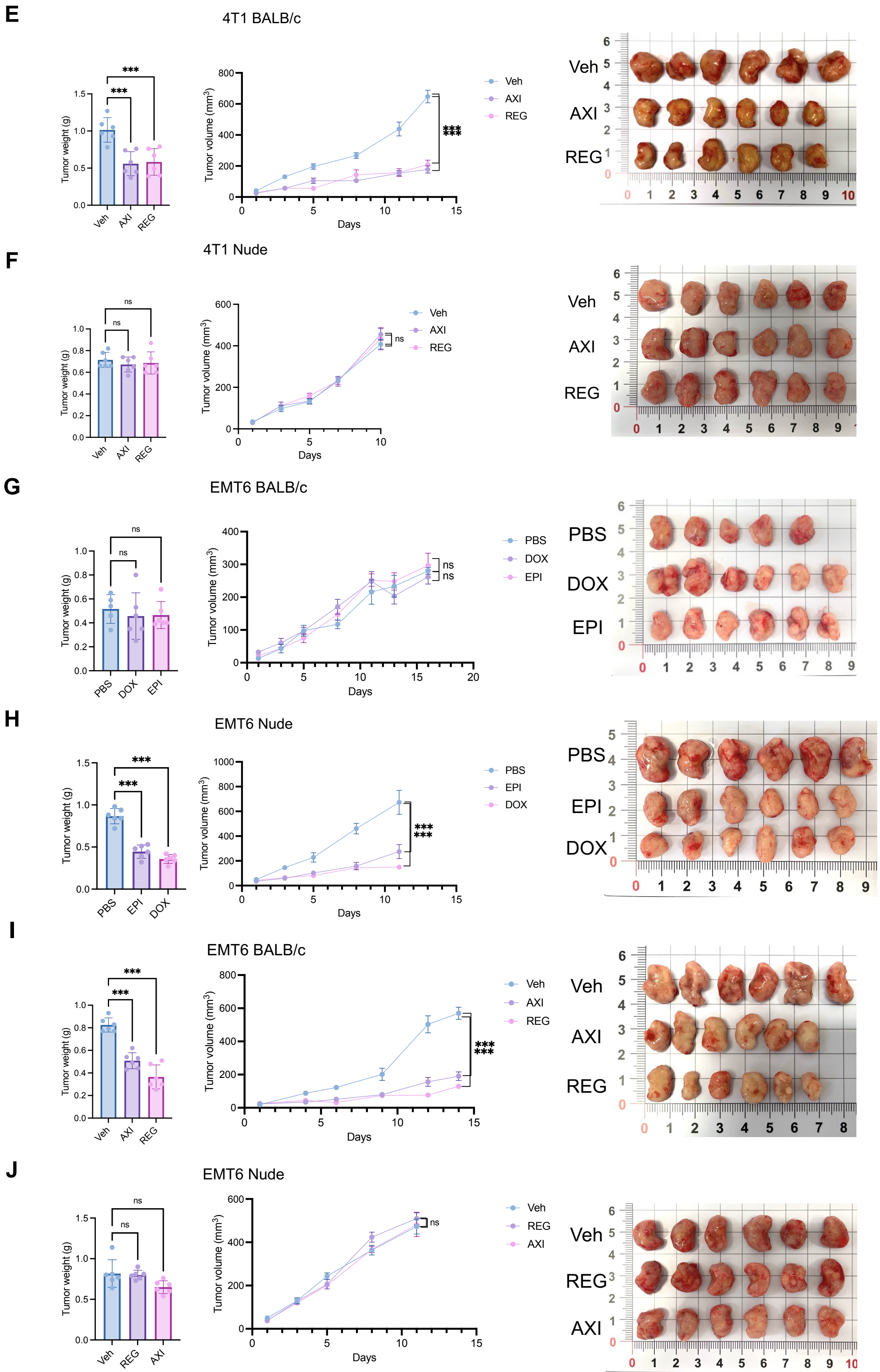

E
4T1 BALB/c
Tumor weight (g)
Veh
AXI
REG
***
***
Tumor volume (mm3)
Days
***
Veh
AXI
REG
F
4T1 Nude
Tumor weight (g)
ns
ns
Tumor volume (mm3)
Days
ns
Veh
AXI
REG
G
EMT6 BALB/c
Tumor weight (g)
PBS
DOX
EPI
ns
ns
Tumor volume (mm3)
Days
ns
ns
PBS
DOX
EPI
H
EMT6 Nude
Tumor weight (g)
PBS
EPI
DOX
***
***
Tumor volume (mm3)
Days
***
***
PBS
EPI
DOX
I
EMT6 BALB/c
Tumor weight (g)
Veh
AXI
REG
***
***
Tumor volume (mm3)
Days
***
***
Veh
AXI
REG
J
EMT6 Nude
Tumor weight (g)
Veh
REG
AXI
ns
ns
Tumor volume (mm3)
Days
ns
Veh
REG
AXI

Supplementary Figure 4. Drug responses are affected by TIME.

(A) Tumor growth and body weight curves of 4T1 cells implanted in BALB/c mice and treated with epirubicin (EPI), gemcitabine (GEM) or paclitaxel (PAC) ($n \geq 5$). (B) Representative images and weight comparisons of tumors and spleens from BALB/c mice implanted with 4T1 cells and treated with EPI, GEM or PAC ($n \geq 5$). (C) Tumor growth and body weight curves of 4T1 cells implanted in Nude mice and treated with EPI, GEM or PAC ($n \geq 5$). (D) Representative images and weight comparison of tumors and spleens from Nude mice implanted with 4T1 cells and treated with EPI, GEM or PAC ($n \geq 5$). (E) Tumor growth curves, tumor weight and representative images of tumors from 4T1 cell-bearing BALB/c mice treated with AXI or REG ($n = 6$). (F) Tumor growth curves, tumor weight and representative images of tumors from 4T1 cell-bearing Nude mice treated with AXI or REG ($n = 6$). (G) Tumor growth curves, tumor weight and representative images of tumors from EMT6 cell-bearing BALB/c mice treated with DOX or EPI ($n \geq 5$). (H) Tumor growth curves, tumor weight and representative images of tumors from EMT6 cell-bearing Nude mice treated with DOX or EPI ($n = 6$). (I) Tumor growth, tumor weight and representative images of tumors from EMT6 cell-bearing BALB/c mice treated with AXI or REG ($n = 6$). (J) Tumor growth, tumor weight and representative images of tumors from EMT6 cell-bearing Nude mice treated with AXI or REG ($n = 6$). Data are presented as mean ± SEM; ns, not significant; *p<0.05; **p<0.01; ***p<0.001; ****p<0.0001.

A

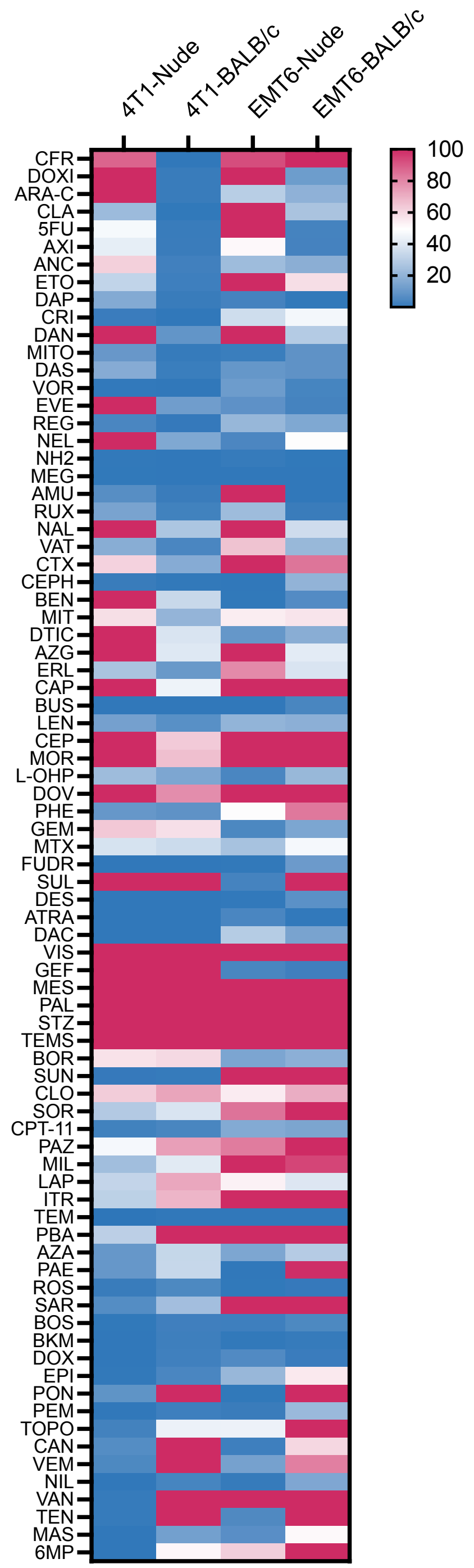

B

Drugs perform better in MTC with an intact TIME

Pharmacologic Class	Drug Name
Kinase Inhibitors	Axitinib
	Crizotinib (PF-02341066)
	Danusertib (PHA-739358)
	Dasatinib
	Regorafenib (BAY 73-4506)
	Amuvatinib (MP-470)
	Ruxolitinib (INCB018424)
	Vatalanib
Antimetabolites	Carmofur
	Doxifluridine
	Cytarabine
	Cladribine
	Adrucil (Fluorouracil)
	Ancitabine hydrochloride
	Nelarabine (Arranon)
Topoisomerase Inhibitors	Etoposide (VP-16)
	Mitoxantrone
Alkylating Agent	Mechlorethamine HCL
Epigenetic Agent	Vorinostat (SAHA)
mTOR Inhibitor	Everolimus (RAD001)
Hormonal Therapy	Megestrol Acetate
Other Mechanism	DAPT (GSI-IX)

C

Drugs perform better in MTC deficient in T cells

Pharmacologic Class	Drug Name
Kinase Inhibitors	Saracatinib
	Bosutinib (SKI-606)
	BKM120 (Buparlisib)
	Ponatinib (AP24534)
	Vemurafenib (PLX4032)
	Nilotinib (AMN-107)
	Vandetanib (Zactima)
	Masitinib
Chemotherapy - DNA Targeting	
Topoisomerase Inhibitors	Doxorubicin (Adriamycin)
	Epirubicin hydrochloride
	Topotecan hydrochloride
	Teniposide
Antimetabolites	Azacitidine (Vidaza)
	Pemetrexed disodium
	Mercaptopurine
Alkylating Agent	Chlorambucil
PPAR-γ Agonist	Rosiglitazone (Avandia)
Protein Phosphatase Inhibitor	Cantharidin
Natural Product	Paeoniflorin

Supplementary Figure 5. High-throughput drug screening identifies drug responses in MTC models with or without intact TIME.

(A) Heatmap of IC_{50} following treatment with indicated drugs across 4T1 or EMT6 tumor MTC models derived from BALB/c or Nude mice. (B) Drugs that perform better in MTC with an intact TIME. (C) Drugs that perform better in MTC deficient in T cells.

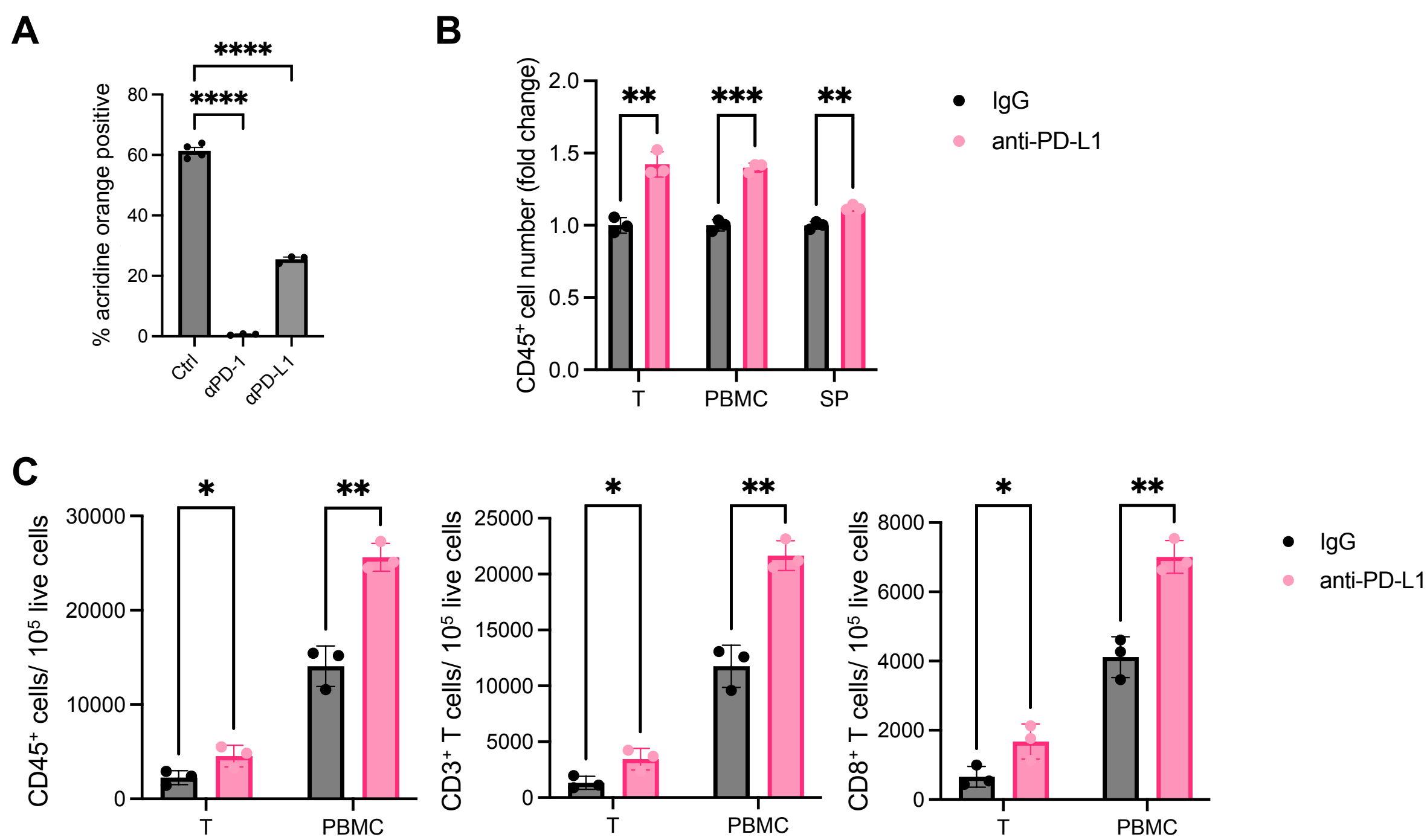

Supplementary Figure 6. MTC model identifies ICB drug effects with or without co-culture with PBMCs or splenocytes.

(A) Quantification of viable cells by AOPI staining in *Brca1*$^{+/-}$ MTC models treated with the indicated ICB drugs at day 3 (n ≥ 3). (B) PBMCs or splenocytes were co-cultured with Hepa1-6 tumor tissues in the MTC system. Quantification of fold changes in $CD45^+$ cell numbers treated with IgG or anti-PD-L1 (n = 3). T, tumor tissues alone; PBMC, MTC co-culture with PBMCs; SP, MTC co-culture with splenocytes. (C) Quantification of $CD45^+$, $CD3^+$ T cell and $CD8^+$ T cell numbers among 10^5 live cells in a human NSCLC sample (NSCLC-4) MTC model, co-cultured with or without autologous PBMCs, following treatment with IgG or anti-PD-L1 (n = 3). Data are presented as mean ± SD; *p<0.05; **p<0.01; ***p<0.001; ****p<0.0001.

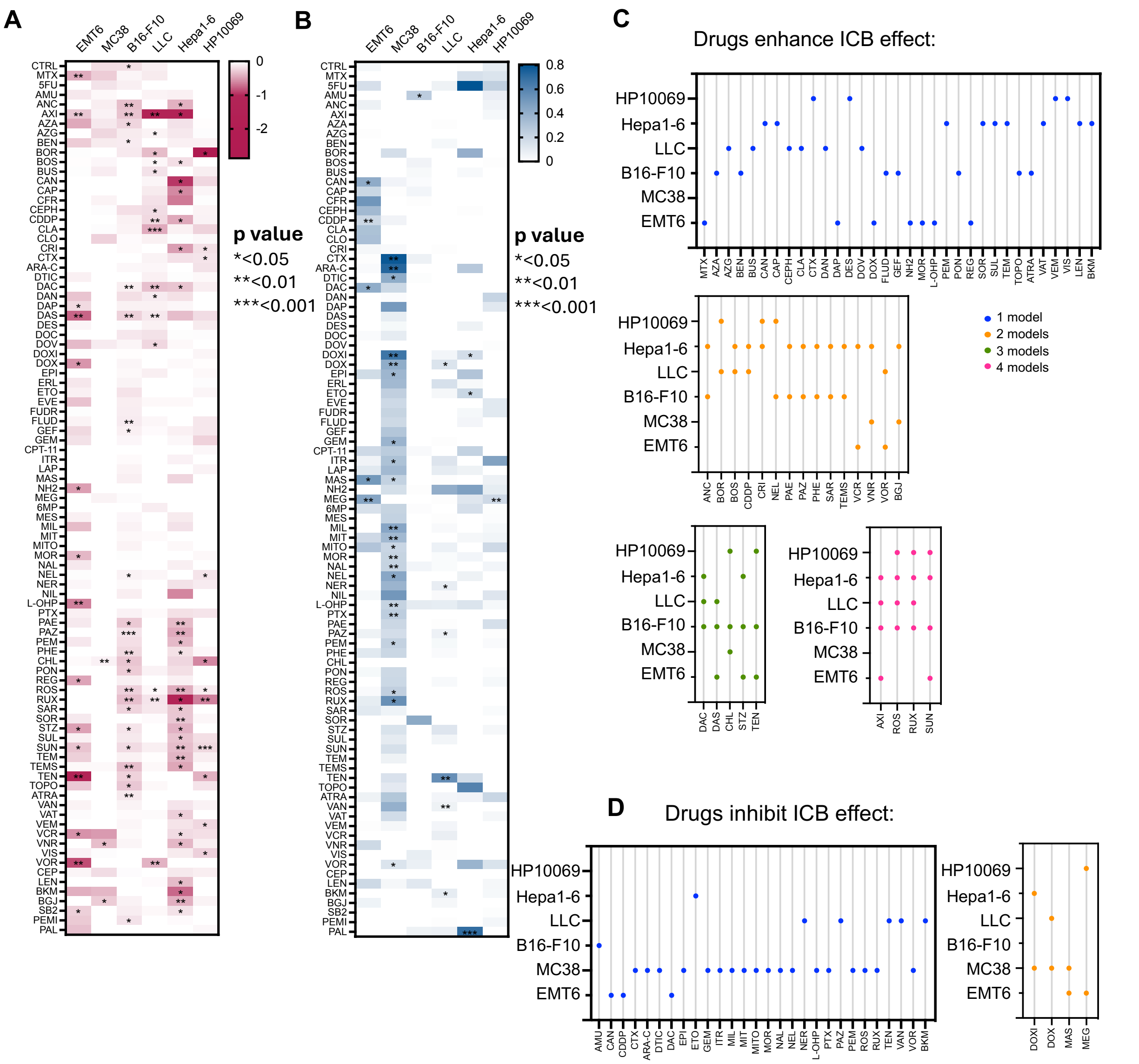

Supplementary Figure 7. High-throughput screening in the MTC model identifies synergistic drug combinations with immune checkpoint blockade.

(A) Heatmap of the log fold change (values < 0) in cell viability following treatment with anti-PD-L1 compared to IgG control across tumor MTC models combined with the indicated drugs. (B) Heatmap of the log fold change (values > 0) in cell viability following treatment with anti-PD-L1 compared to IgG control across tumor MTC models combined with the indicated drugs. (C) Summary of drugs shown enhanced treatment effect combined with anti-PD-L1 among different tumor MTC models. (D) Summary of drugs shown reduced treatment effect combined with anti-PD-L1 among different tumor MTC models. Data are presented as mean values; *p<0.05; **p<0.01; ***p<0.001.

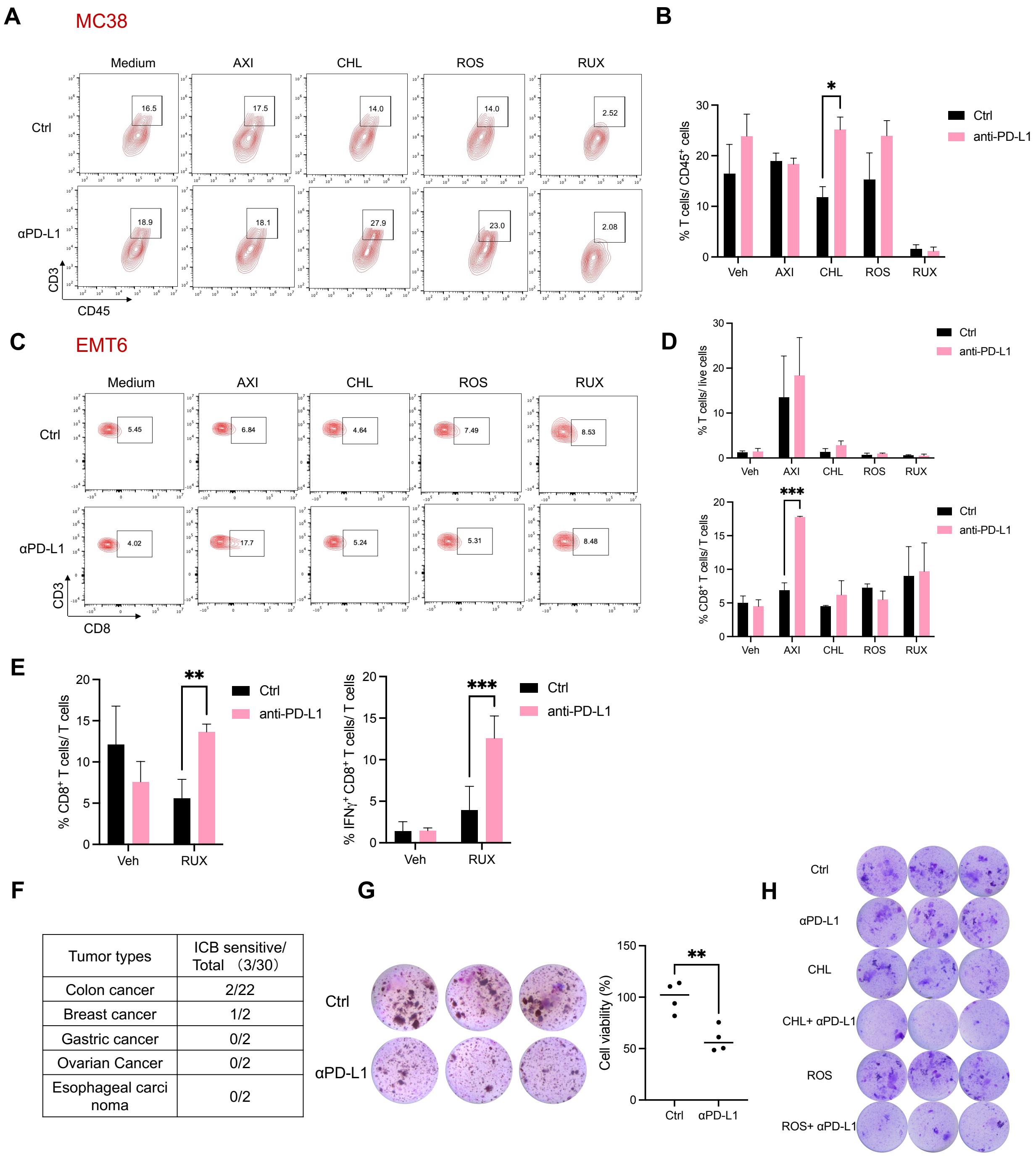

Supplementary Figure 8. Drugs screened from MTC model synergize with immune checkpoint blockade.

(A) Flow cytometry analysis showing the frequency of $CD3^+$ T cells in MC38 MTC model treated with IgG or anti-PD-L1 combined with the indicated drugs. (B) Quantification of T cell population among immune cells in MC38 MTC model treated with IgG or anti-PD-L1 combined with the indicated drugs (n = 3). (C) Flow cytometry analysis showing the frequency of $CD8^+$ T cells in

EMT6 MTC model treated with IgG or anti-PD-L1 combined with the indicated drugs. (D) Quantification of T cell population among live cells and $CD8^+$ T cell population among T cells in EMT6 MTC model treated with IgG or anti-PD-L1 combined with the indicated drugs (n = 3). (E) Quantification of $CD8^+$ T cell and $IFN\gamma^+$ $CD8^+$ T cell population among T cells in Hepa1-6 MTC model treated with IgG or anti-PD-L1 combined with the indicated drugs (n = 3). (F) A table summarized the efficacy of IgG or anti-PD-L1 treatment across various human cancer types. (G) (Left) Representative images of MTT assay in a human colon sample treated with IgG or anti-PD-L1. (Right) Corresponding quantification of cell viability under each condition (n = 4). (H) Representative images of MTT assay in a human colon cancer sample treated with IgG or anti-PD-L1 combined with the indicated drugs. Data are presented as mean ± SD; *$p<0.05$; **$p<0.01$; ***$p<0.001$.

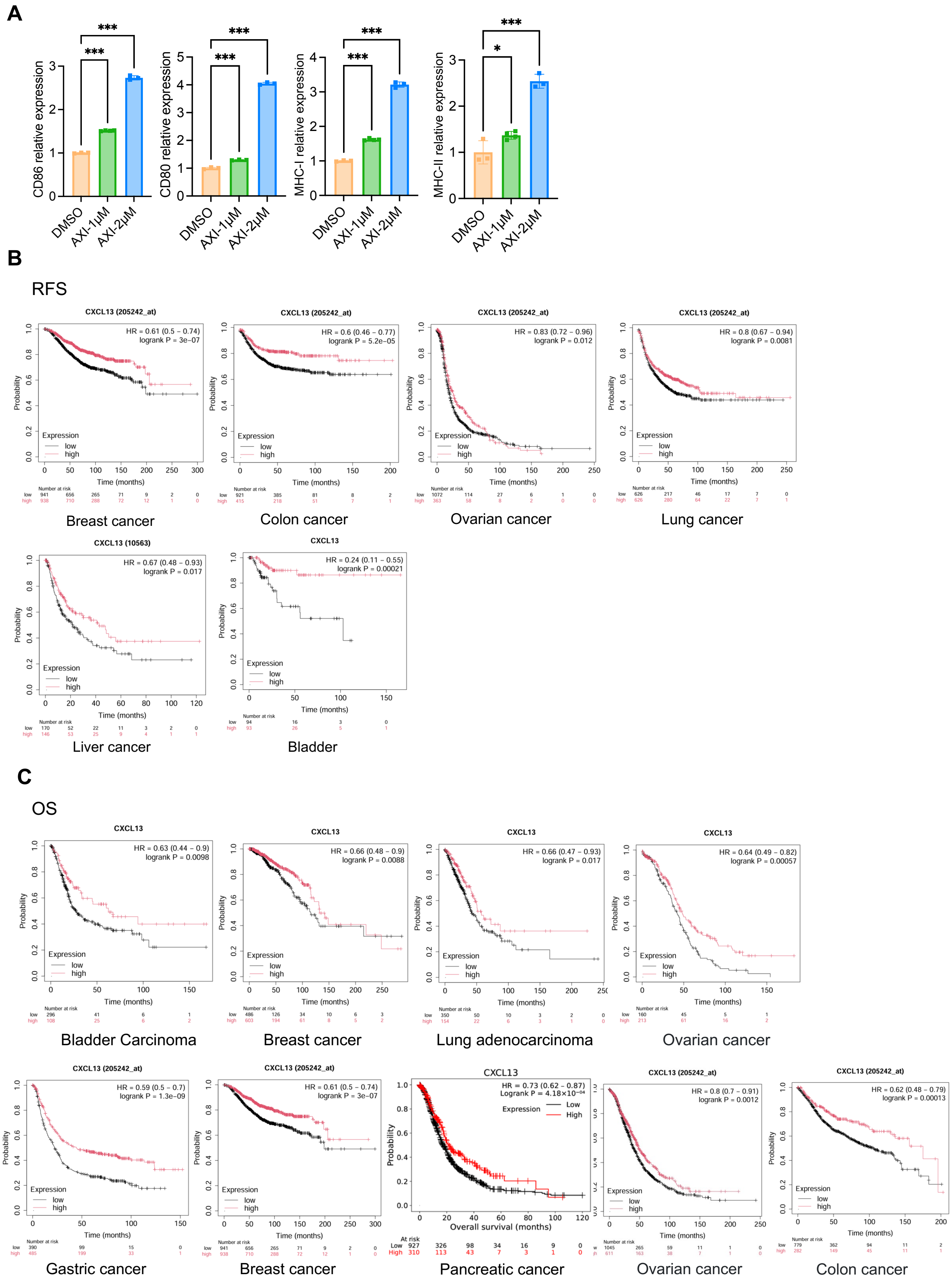

A
CD86 relative expression
CD80 relative expression
MHC-I relative expression
MHC-II relative expression
DMSO
AXI-1μM
AXI-2μM
***
*
B
RFS
CXCL13 (205242_at)
CXCL13 (10563)
CXCL13
HR = 0.61 (0.5 − 0.74)
logrank P = 3e−07
HR = 0.6 (0.46 − 0.77)
logrank P = 5.2e−05
HR = 0.83 (0.72 − 0.96)
logrank P = 0.012
HR = 0.8 (0.67 − 0.94)
logrank P = 0.0081
HR = 0.67 (0.48 − 0.93)
logrank P = 0.017
HR = 0.24 (0.11 − 0.55)
logrank P = 0.00021
Probability
Time (months)
Expression
low
high
Breast cancer
Colon cancer
Ovarian cancer
Lung cancer
Liver cancer
Bladder
C
OS
HR = 0.63 (0.44 − 0.9)
logrank P = 0.0098
HR = 0.66 (0.48 − 0.9)
logrank P = 0.0088
HR = 0.66 (0.47 − 0.93)
logrank P = 0.017
HR = 0.64 (0.49 − 0.82)
logrank P = 0.00057
HR = 0.59 (0.5 − 0.7)
logrank P = 1.3e−09
HR = 0.61 (0.5 − 0.74)
logrank P = 3e−07
HR = 0.73 (0.62 − 0.87)
HR = 0.8 (0.7 − 0.91)
logrank P = 0.0012
HR = 0.62 (0.48 − 0.79)
logrank P = 0.00013
Overall survival (months)
At risk
Low
High
Bladder Carcinoma
Breast cancer
Lung adenocarcinoma
Ovarian cancer
Gastric cancer
Breast cancer
Pancreatic cancer
Ovarian cancer
Colon cancer

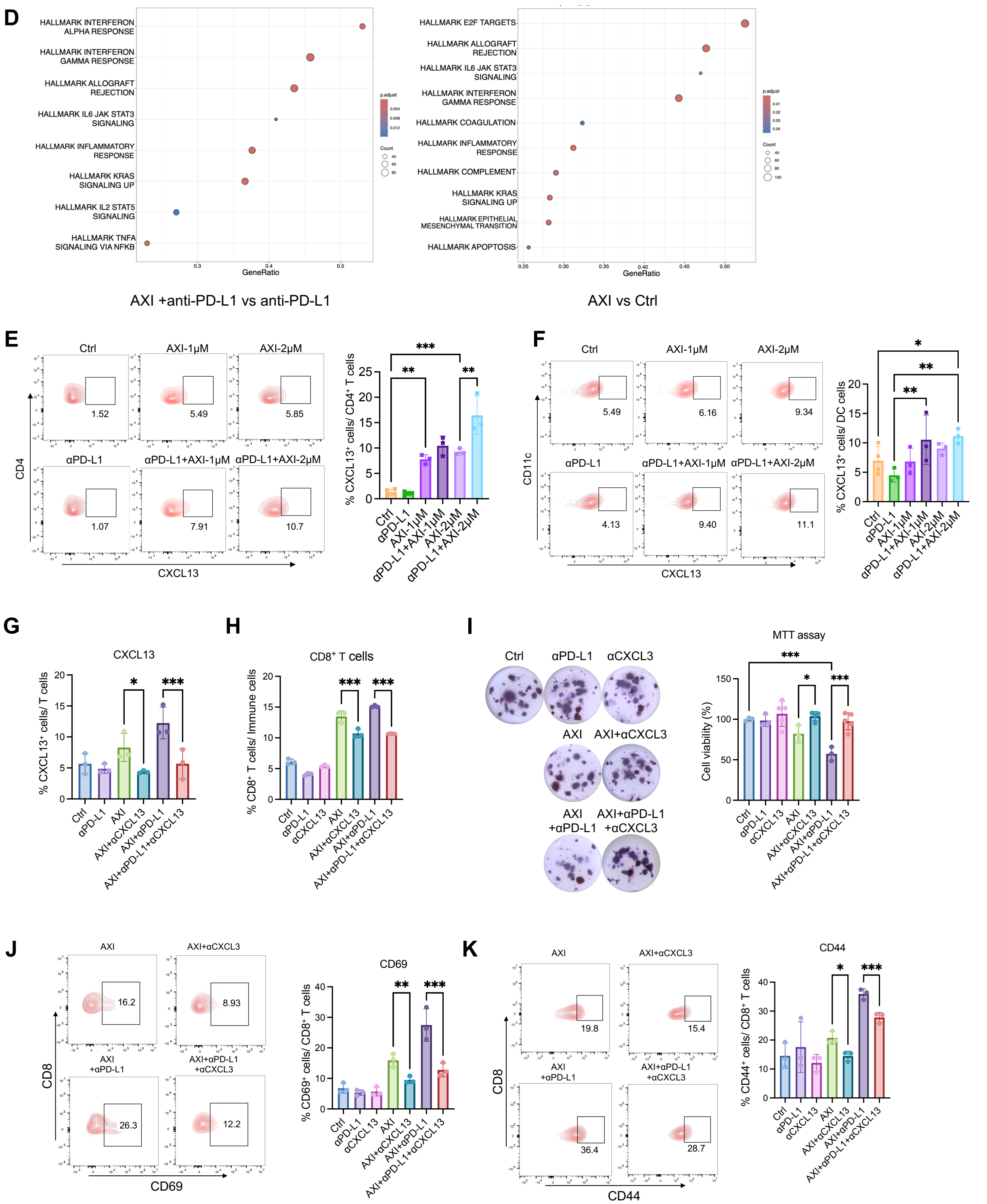

Supplementary Figure 9. Axitinib potentiates ICB efficacy by enhancing antigen presentation and inducing CXCL13-mediated T cell recruitment.

(A) Quantification of CD80, CD86, MHC-I and MHC-II expression in DC2.4 cells treated with 1

or 2 μM AXI (n ≥ 3). (B) Kaplan–Meier analysis of relapse-free survival (RFS), stratified by CXCL13 expression (low versus high) across multiple human cancer types. (C) Kaplan–Meier analysis of overall survival (OS), stratified by CXCL13 expression (low versus high) across multiple human cancer types. Data were analyzed using the Kaplan-Meier Plotter database (http://kmplot.com/). (D) Gene set enrichment analysis (GSEA) of RNA-seq data from Hepa1-6–derived MTC models treated with anti-PD-L1 alone or in combination with AXI. (E) (Left) Flow cytometry analysis of CXCL13$^+$ cell proportion among CD4$^+$ T cells in Hepa1-6 MTC model after treatment with anti-PD-L1 alone or combined with AXI. (Right) Quantification of CXCL13$^+$-producing CD4$^+$ T cells after treatment with anti-PD-L1 alone or combined with AXI (n ≥ 3). (F) (Left) Flow cytometry analysis of CXCL13$^+$ cell proportion among dendritic cells in Hepa1-6 MTC model after treatment with anti-PD-L1 alone or combined with AXI. (Right) Quantification of CXCL13$^+$-producing dendritic cells after treatment with anti-PD-L1 alone or combined with AXI (n ≥ 3). (G) FACS quantification of CXCL13$^+$ cell proportion among T cells within EMT6 MTC model under control conditions, treatment with anti-PD-L1 or AXI alone, anti-PD-L1 combined with AXI, or anti-PD-L1 plus AXI in the presence of a CXCL13-neutralizing antibody (n = 3). (H) FACS quantification of CD8$^+$ T cells among immune cells in EMT6 MTC model under control conditions, treatment with anti-PD-L1 or AXI alone, anti-PD-L1 combined with AXI, or anti-PD-L1 plus AXI in the presence of a CXCL13-neutralizing antibody (n = 3). (I) Representative images of MTT assay and corresponding cell viability in EMT6 MTC models under control conditions, treatment with anti-PD-L1 or AXI alone, anti-PD-L1 combined with AXI, or anti-PD-L1 plus AXI in the presence of a CXCL13-neutralizing antibody (n ≥ 3). (J) (Left) Flow cytometry analysis of CD69$^+$ cell proportion among CD8$^+$ T cells and (Right) corresponding quantification in EMT6 MTC models under control conditions, treatment with anti-PD-L1 or AXI alone, anti-PD-L1 combined with AXI, or anti-PD-L1 plus AXI in the presence of a CXCL13-neutralizing antibody (n = 3). (K) (Left) Flow cytometry analysis of CD44$^+$ cell proportion among CD8$^+$ T cells and (Right) corresponding quantification in EMT6 MTC models under control conditions, treatment with anti-PD-L1 or AXI alone, anti-PD-L1 combined with AXI, or anti-PD-L1 plus AXI in the presence of a CXCL13-neutralizing antibody (n = 3). Data are presented as mean ± SD; *p<0.05; **p<0.01; ***p<0.001.

Supplementary Table 1. Drug list for high-throughput screening.

Abbreviation	Drug names	Drug concentrations (μM)
MTX	Abitrexate (Methotrexate)	5
5FU	Adrucil (Fluorouracil)	20
AMU	Amuvatinib (MP-470)	20
ANC	Ancitabine hydrochloride	5
AXI	Axitinib	5
AZA	Azacitidine (Vidaza)	20
AZG	Azaguanine-8	10
BEN	Bendamustine hydrochloride	20
BOR	Bortezomib	0.1
BOS	Bosutinib (SKI-606)	5
BUS	Busulfan	20
CAN	Cantharidin	20
CAP	Capecitabine (Xeloda)	20
CFR	Carmofur	10
CEPH	Cephalomannine	5
CDDP	Cisplatin	5
CLA	Cladribine	5
CLO	Clofarabine	10
CRI	Crizotinib (PF-02341066)	10
CTX	Cyclophosphamide monohydrate	20
ARA-C	Cytarabine	5
DTIC	Dacarbazine (DTIC-Dome)	20
DAC	Dacomitinib (PF299804,PF-00299804)	5
DAN	Danusertib (PHA-739358)	20
DAP	DAPT (GSI-IX)	20
DAS	Dasatinib	20
DES	Diethylstilbestrol (Stilbestrol)	20
DOC	Docetaxel (Taxotere)	0.1
DOV	Dovitinib (TKI-258)	20
DOXI	DOXIFLURIDINE	20
DOX	Doxorubicin (Adriamycin)	2
EPI	Epirubicin hydrochloride	2
ERL	Erlotinib hydrochloride	5
ETO	Etoposide (VP-16)	5
EVE	Everolimus (RAD001)	10
FUDR	Floxuridine	5

FLUD	Fludarabine (Fludara)	10
GEF	Gefitinib (Iressa)	10
GEM	Gemcitabine Hydrochloride	0.1
CPT-11	Irinotecan	20
ITR	Itraconazole (Sporanox)	20
LAP	Lapatinib Ditosylate (Tykerb)	20
MAS	Masitinib	20
NH2	Mechlorethamine HCL	20
MEG	Megestrol acetate	20
6MP	Mercaptopurine	20
MES	Mesna (Uromitexan, Mesnex)	20
MIL	Miltefosine (hexadecyl phosphocholine)	20
MIT	Mitotane (Lysodren)	5
MITO	Mitoxantrone	5
MOR	Moroxydine	5
NAL	Naloxone HCl	20
NEL	Nelarabine (Arranon)	20
NER	Neratinib	20
NIL	Nilotinib (AMN-107)	20
L-OHP	Oxaliplatin (Eloxatin)	20
PTX	Paclitaxel (Taxol)	0.1
PAE	Paeoniflorin	20
PAZ	Pazopanib hydrochloride	20
PEM	Pemetrexed disodium	10
PHE	Phenylbutazone (Butazolidin, Butatron)	20
CHL	Chlorambucil	20
PON	Ponatinib (AP24534)	20
REG	Regorafenib (BAY 73-4506)	20
ROS	Rosiglitazone (Avandia)	20
RUX	Ruxolitinib (INCB018424)	20
SAR	Saracatinib	10
SOR	Sorafenib (Nexavar)	10
STZ	Streptozotocin (Zanosar)	20
SUL	Sulindac (Clinoril)	20
SUN	Sunitinib Malate (Sutent)	10
TEM	Temocapril hydrochloride	10
TEMS	Temsirolimus (Torisel)	10
TEN	Teniposide	10
TOPO	Topotecan hydrochloride	5

ATRA	Tretinoin (Aberela)	10
VAN	Vandetanib (Zactima)	20
VAT	Vatalanib	20
VEM	Vemurafenib (PLX4032)	20
VCR	Vincristine	1
VNR	Vinorelbine (Navelbine)	1
VIS	Vismodegib (GDC-0449)	2
VOR	Vorinostat (SAHA)	10
CEP	Cepharanthine	10
LEN	Lenvatinib	10
BKM	BKM120	5
BGJ	BGJ398	10
SB2	SB2220000	5
PEMI	Pemigatinib	20
PAL	Palbociclib	10

Supplementary Table 2. Clinical and molecular characteristics of patient-derived tumors and their functional responses to ICB in the MTC Platform.

Sample ID	Atezolizumab response in MTC	Primary Site	Pathological Diagnosis	TNM Staging	MMR/MSI Status	PD-L1 Expression (22C3)	Key Gene Mutations / Biomarkers
ESCA-1	No	Esophageal	Squamous Cell Carcinoma	ypT2N0Mx	pMMR	N/A	p16-
ESCA-2	No	Esophageal	Squamous Cell Carcinoma	pT3N1Mx	N/A	CPS > 10	No
ESCA-3	No	Esophageal	Squamous Cell Carcinoma	ypT3N0Mx	N/A	1 < CPS < 10	EGFR(5B7) > 90% moderate to strong; HER2(4B5) +
ESCA-4	No	Esophageal	Squamous Cell Carcinoma	pT1bN3Mx	pMMR	CPS ≈ 10	p53+(mutant); EGFR(5B7) > 90% moderate to strong
ESCA-5	No	Esophageal	Squamous Cell Carcinoma	pT2N0Mx	N/A	CPS ≈ 20	EGFR(5B7) ≈ 80% moderate to strong
GC-1	No	Gastric	Adenocarcinoma	pT3N3aMx	pMMR	CPS ≈ 30	EGFR(5B7) ≈ 90% weak to moderate
GC-2	No	Gastric	Adenocarcinoma	pT4aN2Mx	pMMR	1 < CPS < 10	EGFR(5B7) ≈ 90% weak to moderate
GC-3	No	Gastric	Adenocarcinoma	pT1bN2Mx	MLH1–, MSH2+, MSH6+, PMS2–	CPS ≈ 35	EGFR(5B7) ≈ 90% moderate to strong
GC-4	No	Gastric	Adenocarcinoma	pT2N0Mx	pMMR	CPS ≈ 8	EGFR(5B7) ≈ 90% weak to moderate; HER2(4B5) 2+
GC-5	No	Gastric	Adenocarcinoma	pT3N0Mx	pMMR	CPS ≈ 10	EGFR(5B7) ≈ 30% weak
GC-6	No	Gastric	Adenocarcinoma	pT3N2Mx	pMMR	CPS ≈ 17	No
GC-7	No	Gastric	Mixed Carcinoma	pT3N0Mx	pMMR	1 < CPS < 10	EGFR(5B7) ≈ 60% weak; HER2(4B5) 2+

CT-1	No	Colorectal	Adenocarcinoma	pT3N0Mx	pMMR	CPS ≈ 8	EGFR(5B7) ≈ 60% weak to moderate
CT-2	No	Colorectal	Adenocarcinoma	pT1N0Mx	pMMR	CPS ≈ 2	EGFR(5B7) ≈ 80% weak to moderate
CT-3	No	Colorectal	Adenocarcinoma	pTisN0Mx	pMMR	N/A	HER2(4B5) ≈50% 1+
CT-4	No	Colorectal	Adenocarcinoma	pT3N1cMx	MLH1+, MSH2−, MSH6−, PMS2+	CPS > 10	EGFR(5B7) > 90% weak, HER2(4B5) ≈ 5% 1+
CT-5	No	Colorectal	Adenocarcinoma	N/A	N/A	N/A	N/A
CT-6	No	Colorectal	Adenocarcinoma	T4aN2aM0	pMMR	N/A	No
CT-7	No	Colorectal	Adenocarcinoma	pT3N1bMx	pMMR	1 < CPS < 10	EGFR(5B7) > 90% moderate to strong; HER2(4B5) ≈ 30% 1+
CT-8	No	Colorectal	Adenocarcinoma	ypT3N0Mx	pMMR	1 < CPS < 10	No
CT-9	Yes	Colorectal	Adenocarcinoma	pT3N1bMx	pMMR	CPS > 10	EGFR(5B7) ≈ 80% weak to moderate; HER2(4B5) ≈ 35% 1+
CT-10	Yes	Colorectal	Adenocarcinoma	pT3N0Mx	pMMR	CPS > 50, TPS ≈ 70%	HER2(4B5) ≈ 35% 1+
